# Potential Antidote Against Lead Toxicity—A Review

**DOI:** 10.3390/ijms27114881

**Published:** 2026-05-28

**Authors:** Zuzanna Romanowska, Agnieszka Ścibior, Lidia Radko, Tomasz Męcik-Kronenberg

**Affiliations:** 1 Department of Paediatrics, Medical University of Warsaw, Żwirki i Wigury St. 63A, 02-091 Warsaw, Poland; zuzannamikus@gmail.com; 2 Laboratory of Oxidative Stress, Department of Biomedicine and Environmental Research, Institute of Biological Sciences, Faculty of Medicine, The John Paul II Catholic University of Lublin, Konstantynów St. 1J, 20-708 Lublin, Poland; 3 Department of Preclinical Sciences and Infectious Diseases, Faculty of Veterinary Medicine and Animal Sciences, Poznan University of Life Sciences, Wolynska St. 35, 60-637 Poznan, Poland; lidia.radko@up.poznan.pl; 4 Department of Pathomorphology, Faculty of Medical Sciences in Zabrze, Medical University of Silesia, 3 Maja St. 13, 41-800 Zabrze, Poland; patolog@interia.pl; 5 Collegium Medicum im. Dr. Władysław Biegański, Jan Długosz University, Washington St. 4/8, 42-200 Czestochowa, Poland

**Keywords:** animals, antioxidants, lead toxicity, occupational/environmental lead exposure

## Abstract

As chronic lead (Pb) exposure is a serious threat to public health in many countries, we intended to provide a review of the effective antidote for Pb intoxication. In this context, we explored the recent literature focusing on animal studies that provide clear evidence of the efficacy of supplementation with certain antioxidants, such as spirulina, curcumin, garlic, vitamin C, and vitamin E, for different periods of time, in the conditions of Pb exposure, drawing attention to the mechanisms of their beneficial action. Data collected clearly indicate that spirulina, curcumin, garlic, vitamin C, and vitamin E may play an important role in alleviating some of the toxic effects caused by Pb. Their use may represent an interesting direction in the development of new therapies to combat the toxicity of this toxic, non-essential metal. However, it seems essential to identify a safe dose range for these antioxidants in order to derive benefits from their properties during Pb intoxication. Clinical trials seem to be necessary to recognize the long-term effects of supplementation with these antioxidants and to establish the minimum effective dose with minimal side effects. Future studies are also required to elucidate the molecular mechanisms of action of spirulina, curcumin, garlic, vitamin C, and vitamin E against Pb toxicity and the mechanisms of their interactions with chelating agents to enhance potential beneficial effects.

## 1. Introduction

### 1.1. Lead—Background

Lead (chemical symbol Pb) is a soft and malleable metal with an atomic number of 82. Freshly cut, it is whitish-blue, and tarnishes to a dull gray in the air. It is located in period 6 and group 14 of the periodic table. Lead’s high density, low melting point, conductivity, and relatively low susceptibility to oxidation account for its usefulness. Considering its non-biodegradable nature and continuous use, its concentration and accumulation in the environment have been an ongoing issue for years, until now. This condition is caused by continuous use of Pb in many industries [1], as listed further in this article. Depending on the level of country development, Pb sources and Pb use in general vary from dietary exposure (food, soil, drinking water, contaminated spices included) [2,3,4,5,6], industrial fumes, improper battery recycling [7] in low and middle-income countries to gasoline, pipes, and paints in highly developed regions of the world [8].

Although the incidence of Pb poisoning has declined in recent years, especially in high-income countries, the problem still persists in some regions of low- and middle-income countries. It may be associated with the introduction of more efficient and precise regulations—for example, in the U.S., Pb-free paints and Pb-free gasoline were introduced by the 1980s [9]. For instance, paints containing this metal are still in use in Nigeria and other developing countries [10]. According to Zhou et al. [11], China, India, and Bangladesh were the top three countries with the largest number of deaths and DALYs (disability-adjusted life years) due to Pb exposure in 2019. In 2021, the Centers for Disease Control and Prevention changed the blood Pb reference value in children to 3.5 μg/dL, whereas in the 1960s, the same organization proposed 60 μg/dL as the threshold [12].

### 1.2. Absorption, Distribution, and Excretion of Pb—The Most Important Aspects

Pb enters the human body in a few ways, i.e., through the gastrointestinal tract, respiratory system, and skin [13,14]. Absorption of this metal primarily occurs in the duodenum and depends on a variety of factors, including the particle size, route of exposure, nutritional status, health, and age of individuals [14]. Pb, which utilizes channels or transporters of other elements such as calcium (Ca), iron (Fe), and zinc (Zn) [15], is absorbed into the body, with children generally absorbing a higher percentage than adults [14]. Children can absorb 40–50% of an oral dose of water-soluble Pb compared to 3–10% for adults [14]. The average intake of Pb by adults is estimated to be between 320 and 440 µg/day in different countries. The estimated amounts of Pb intake with food in Poland are in the range of 50–520 µg/day for adults and 40–350 µg/day for children aged 1–3 years [16]. Inhalation represents a significant exposure pathway, particularly for workers in Pb industries. Approximately 30–50% of inhaled Pb is deposited in the lungs [14], leading to gradual destruction of this tissue. From pathologies such as oxidative stress generating inflammation, increased levels of tumor necrosis factor alpha (TNF-α), nuclear factor kappa-light-chain-enhancer of activated B cells (NF-κB), and interleukin 1 (IL-1) [17] observed at the cellular level to incidence of asthma, allergies [18], and lung cancer [19].

After absorption, Pb accumulates in soft tissues such as liver, kidney, heart, spleen, brain, lung, and muscles, and mineralized tissues, including bone and teeth, where it may be stored in the form of complexes with phosphate for a long period [14,20]. Approximately 95% of Pb accumulates in mineralized tissues [13]. The remaining 5% is stored in soft tissues [21]. In the blood, approximately 94–99% of Pb is incorporated into red blood cells (RBC), while the remaining 1–6% resides in the plasma [14,20,21,22]. In RBC, Pb binds to several intracellular proteins, mainly hemoglobin (Hb) and delta-aminolevulinic acid dehydratase (ALAD) [14,20]. In the plasma, 40–75% of Pb is protein-bound, of which 90% binds to albumin [14,20,23], one of the most abundant proteins in the human circulation, responsible for transport functions [24]. In the plasma, Pb may also interact with other proteins, e.g., transferrin (Tf), gamma-globulin, and metalloprotein (MT) [14,25]. Moreover, Pb may bind to citrate, cysteamine, glutathione, histidine, metalloproteins, and oxylate in the plasma [14]. It may also occur in the free ionized (Pb^2+^) form, although Pb^2+^ represents a small percentage of total Pb in this compartment. Notably, no level of Pb is considered completely safe, even at its low concentrations. In adults, a blood Pb level of 5 µg/dL (0.24 µmol/L) or above is considered elevated, including adults in the workplace [26] and pregnant women [27]; however, it should be considered that during gestation, there is no safe level of Pb exposure. In children, a blood Pb level of 3.5 µg/dL (0.17 µmol/L) or greater requires monitoring [28]. Blood Pb levels of 10 µg/dL (0.48 µmol/L) or higher are considered toxic [29].

**Figure 1:**
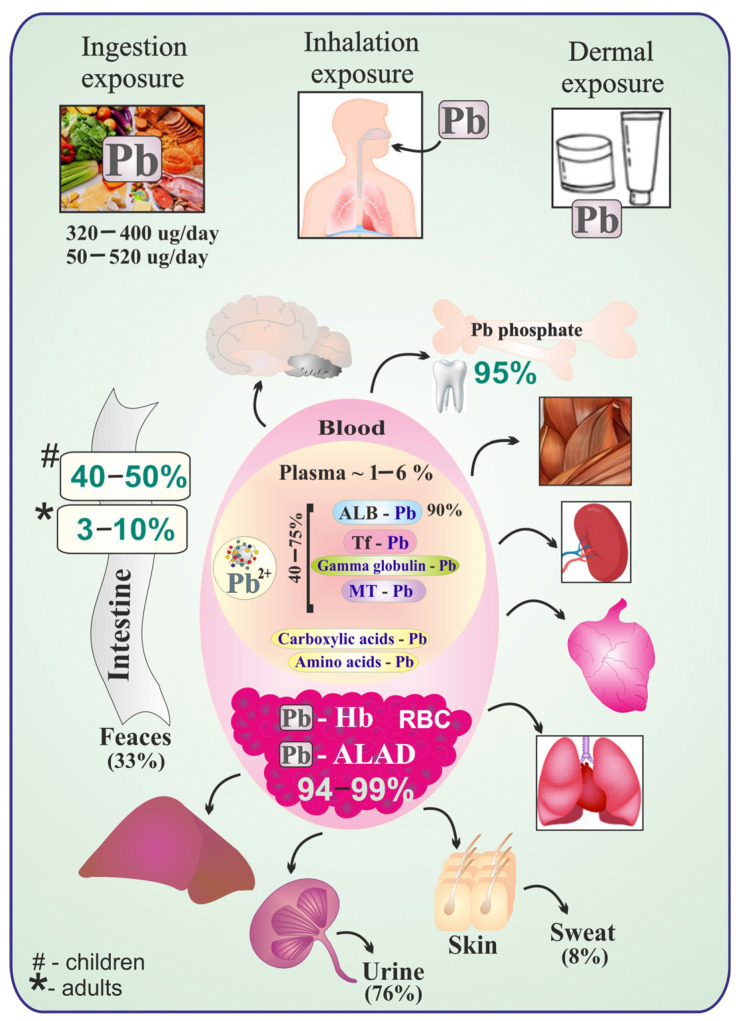
Schematic diagram summarizing absorption, distribution, and excretion of Pb—based on the available literature cited in Section 1.2. ALB: albumin; ALAD: delta-aminolevulinic acid dehydratase; Hb: hemoglobin; MT: metalloprotein; RBC: red blood cells; Tf: transferrin.

Regarding the excretion of Pb, absorbed Pb is filtered through the glomeruli and can accumulate in the renal tubular epithelium [30]. It is primarily excreted in the urine (~76%) [31], while fecal excretion accounts for approximately one-third of total excretion of absorbed Pb (~33%) [14,31]. Small amounts of this metal are excreted through sweat (~8%) [14,31]. A summary of Pb exposure routes, distribution, and excretion is provided in Figure 1.

### 1.3. Environmental Exposure to Pb—A Brief Outline

Although some industrial sources of Pb have been eliminated, excessive environmental exposure to this toxic metal continues to be a public health problem. Many inhabitants of modern cities are chronically exposed to environmental Pb levels. In the general population, which is not occupationally exposed to this xenobiotic, food and beverages are the major sources of exposure [31]. Additionally, airborne Pb particles from burning Pb-containing materials may be inhaled by individuals and markedly raise their blood Pb levels, consequently leading to poisoning with this toxic metal. Depending on the level of exposure, the time required to remove Pb from the body varies. The greater the exposure, the longer the time needed for Pb to leave the body and for health improvement to occur. The amount of time necessary for health improvement may range from months to years, depending on each specific case and on the level of previous Pb exposure [32].

**Figure 2:**
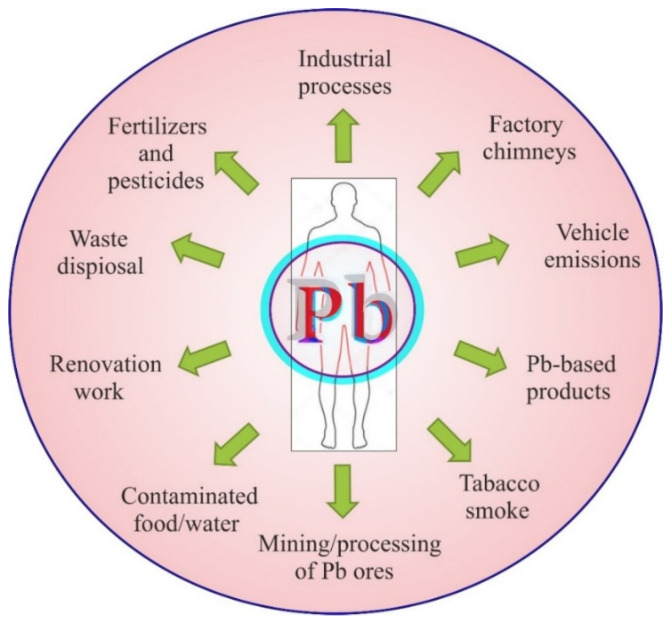
Environmental exposure to Pb—based on the available literature cited in Section 1.3.

The development of industrialization has had an impact on constant Pb pollution. The standards of this metal in the air changed over time and differ depending on ambient outdoor air or workplace exposure. The latter are much higher as they apply to occupational settings. In 1978, OSHA (Occupational Safety and Health Administration) workplace standard lowered the permissible exposure limit (PEL) from 200 µg/m^3^ to 50 µg/m^3^ [33]. Historically established Pb limits for ambient outdoor air ranged from 1.5 µg/m^3^ in 1978, and were then reduced to 0.15 µg/m^3^ in 2008 [34]. The current threshold for this element in the air—0.15 µg/m^3^—was set by the U.S. EPA (U.S. Environmental Protection Agency) in 2016, averaged over a 3-month period [35]. In comparison, in the EU, the actual limit value is 0.5 µg/m^3^, averaging over a 1-year period [36]. According to Patocka and Cerny [37], in industrial regions, the amount of this metal in the atmosphere ranges from 0.2 to 5.0 μg/m^3^. Air, water, and soil are mainly polluted by industrial emissions from mining, fossil fuel burning, and manufacturing. Furthermore, Pb can still be found in human households, especially in wall paints, toys, cosmetics, and pipes [31,38,39]. The most important environmental sources of exposure to Pb involve industrial activities, factory chimneys, vehicle emissions, waste disposal, mining and processing of Pb ores, fertilizers and pesticides, Pb-based products, renovation work, contaminated food and water, and tobacco smoking [1,40,41]. The latter is a significant public health concern due to its association with various health risks [42]. It has been reported that a single cigarette contains 0.6–2.0 µg of Pb [43]. Environmental Pb sources are summarized in Figure 2.

It should be highlighted that children are especially susceptible to Pb exposure, even to its small doses through ingestion, particularly during the first two years of life, when they develop mouthing behaviors and increased mobility. Children’s tissues are also more prone to greater accumulation and absorption of this metal (especially bones and blood). Iron deficiency, which is a common condition in childhood, may even promote Pb absorption [44]. Blood Pb concentrations in children living in Pb-contaminated environments typically increase rapidly between 6 and 12 months of age, peak between 18 and 36 months of age, and then gradually decrease [45]. Based on current knowledge, the WHO has targeted blood Pb concentrations of 5 μg/dL or less for children; however, adverse effects of Pb exposure may occur at levels below this threshold [46]. In 2012, the US National Toxicology Program of the National Institutes of Health reported that, after accounting for other risk factors, blood Pb concentrations < 5 µg/dL (<50 ppb) are strongly linked to intellectual deficits, diminished academic abilities, attention deficits, and behavioral problems (ADHD, irritability, antisocial behaviors) [47]. Concentrations of <10 µg/dL have been proven to cause delayed puberty, impaired postnatal growth, and decreased hearing. As reported by some authors, a higher incidence of delinquency and aggression was found in adolescents with higher bone Pb concentrations [48].

Pb toxicity still remains one of the most frequently studied topics in environmental health. Environmental pollution with this metal and consequent health effects, particularly in children, are still a global problem. Many authors observed unfavorable changes in hematological and biochemical parameters in children with elevated blood Pb (Pb_B_) levels. For example, Li et al. [49] revealed that Pb_B_ levels are associated with decreased erythrocyte (RBC) count, hemoglobin (Hb) level, mean corpuscular hemoglobin (MCH), and platelet (PLT) count in children aged 5–8 years in China. They also found that children with Pb_B_ ≥ 100 µg/L were 2.72, 2.51, and 3.76 times more likely to present with decreased RBC count, Hb level, and PLT count, respectively, compared to those with Pb_B_ ≤ 100 µg/L. Other authors, who examined the relationship between Pb_B_ ≥ 10 μg/dL and anemia in children aged 1–7 years from Lucknow (northern India), demonstrated that elevated Pb_B_ levels (≥10 μg/dL) in children are significantly associated with an increased risk of anemia [50]. They found that, in children with Pb_B_ ≥ 10 μg/dL, the Hb level and hematocrit (Ht) index were lower than in those with Pb_B_ ≤ 10 μg/dL [50]. Rawat et al. [51] also showed a reduced Hb level, Ht index, RBC count, mean corpuscular volume (MCV), MCH, and mean corpuscular hemoglobin concentration (MCHC) as well as an elevated white blood cell (WBC) count in children with Pb_B_ > 10 μg/dL aged 4–12 years from an area with Pb exposure due to the presence of an informal battery recycling unit (India), compared to those with Pb_B_ < 10 μg/dL. The level of Pb_B_ ≥ 10 μg/dL has also been reported by Hegazy et al. [52] to be associated with anemia. Similarly, a decreased Hb level and Ht index, as well as reduced MCV, MCH, and MCHC values, were found by Alvarez-Ortega et al. [53] in children with Pb_B_ 1.7 μg/dL aged 5–16 years from the Paseco Bolivar neighborhood (melting activities) in Cartagena (Colombia). Lowered MCH and MCHC values were also observed by Dai et al. [54] in preschool children with Pb_B_ > 5 μg/dL living in a waste-exposed area in China. Another study, which aimed to assess the association between Pb_B_ and Ht in one to five year-old children living near a primary Pb smelter, showed that the increased risk of decreased Ht in one year-old children was 2% at levels between 0.97 and 1.93 μmol/L (20 and 39 μg/dL), 18% at levels between 1.39 and 2.90 μmol/L (40 and 60 μg/dL), and 40% at Pb_B_ > 2.90 μmol/L (60 μg/dL) [55]. In turn, a decreasing trend in the Hb content with increasing Pb_B_ levels was observed in children aged 3–6 years living in Mumbai (a large industrialized city in India) by Tripathi and co-workers [56]. Other researchers found negative correlations between Pb_B_ levels and Hb content [57] and between Pb_B_ levels and MCV or MCH [58] in children aged 5–9 years living in a tile-glazing area in Ecuador and in children aged 4–15 years old living in Cartagena (Colombia), respectively. A negative association between Pb_B_ levels and Hb, Ht, MCV, and MCH was also noted by Kuang et al. [59] in children aged 7–11 years from Nanjing, an industrial city in eastern China. Jacob et al. [60] reported that an increase in Pb_B_ levels by 10 μg/dL was associated with reduced MCV and MCH in girls aged 5–14 years with Pb_B_ levels in the range of 34–61 μg/dL and >60 μg/dL.

Regarding biochemical parameters, Jin and co-workers [61] demonstrated reduced activity of ALAD in RBC and increased plasma malondialdehyde (MDA) levels, one of the well-known secondary products of lipid peroxidation (LPO) [62], in children aged 3–6 years with Pb_B_ ≥ 100 μg/dL living in Anshan city, a northeast Chinese city famous for its steel refinery. Elevated LPO levels in the blood were also found by Cabral et al. [63] in children with Pb_B_ 0.7 μmol/L (14.5 μg/dL) living near the Mbeubeuss landfill located near Dakar (Senegal) and by Ahamed et al. [64] in children with aplastic anemia, who exhibited significantly higher Pb_B_ levels (9.86 μg/dL) than the control group (4.23 μg/dL). Some components of the antioxidative system have also been observed to be changed. For example, Diouf et al. [65] demonstrated a decrease in the activity of glutathione peroxidase (GPx), accompanied by an increase in the activity of glutathione reductase (GR) and in the glutathione status (GSSG/GSH) in the blood of children living in the urban area of Dakar (Senegal). The mean level of Pb_B_ in these children was significantly higher, i.e., 9.97 μg/dL within a range of 3.10–22 μg/dL, compared to that noted in children from rural regions (5.21 μg/dL). Similarly, Ahamed et al. [64] reported a decreased level of reduced glutathione (GSH) and elevated catalase (CAT) activity in children with aplastic anemia, who exhibited a significantly higher Pb_B_ level (9.86 μg/dL) than the control group (4.23 μg/dL). In turn, Nascimento et al. [66] investigated biomarkers of exposure to ChE inhibitor insecticides and toxic metals/metalloids in children aged five to 16 years living in a rural area of southern Brazil, at two different time points, i.e., period 1, when pesticides were not used in the region, and period 2, when pesticides were extensively used in agriculture. Their research demonstrated elevated activity of GPx and glutathione S-transferase (GST) in period 2, which was probably a compensatory mechanism in response to the increasing exposure to xenobiotics, including Pb.

Hepatic enzymes, which are commonly used clinical biomarkers to assess liver injury and disease, have also been reported to be altered. For example, Rawat et al. [51] reported elevated activity of aspartate aminotransferase (AST) and alkaline phosphatase (ALP) in the serum of Pb-exposed children aged 4–12 years with Pb_B_ < 10 μg/dL/Pb_B_ > 10 μg/dL from an area with informal battery recycling in India. Betanzos-Robledo et al. [67], who conducted a study to estimate the association between cumulative Pb exposure during early life from 1 to 4 years of age and biomarkers of hepatic steatosis in young adulthood in a Mexico City birth cohort, demonstrated associations between cumulative blood Pb levels in early childhood and elevated levels of AST and alanine aminotransferase (ALT) in the blood in young adulthood. The authors concluded that chronic exposure to Pb during childhood is associated with multiple biomarkers of hepatic steatosis in young adulthood in a general-risk Mexican population. Some unfavorable changes in hepatotoxicity biomarkers were also observed in adults chronically exposed to Pb. For example, Chen et al. [68], who investigated blood Pb levels and clinical characteristics of subjects in exposed and reference groups to evaluate the environmental Pb exposure risk to human liver health, found a 2.9-fold increase in the activity of gamma-glutamyl transferase (GGT) in the serum of the exposed individuals, compared to those from the reference group. The concentration of Pb in the exposed group was almost two times higher than in the control group. Pb_B_ levels (27.71 μg/L) and enhanced activity of AST, ALT, ALP, and GGT were also observed by Yan et al. [69] in residents near a mining and smelting area in northwestern China.

Some renal biomarkers essential in assessing kidney function have also been investigated. For example, Bernard et al. [70], who studied renal tubular function in children aged 12–15 years recruited from two schools in the vicinity of a Pb smelter and children of similar age from a school in a rural area, found that children from the polluted areas had higher urinary excretion of retinol binding protein (RBP), the most sensitive biomarker for loss of proximal renal tubule function [71], which paralleled the level of Pb in the blood (girls: 94.4 and 129 μg/L; boys: 109 and 149 μg/L). Verberk et al. [72], who evaluated children residing at various distances from a Pb smelter, demonstrated a relationship between Pb_B_ levels (342 μg/L) and the activity of N-acetyl-beta-D-glucosaminidase (NAG) in urine, suggesting renal tubular damage due to Pb exposure. Increased lactate dehydrogenase (LDH) activity in the urine and proteinuria were noted by Cabral et al. [63] in children with Pb_B_ of 0.7 μmol/L (14.5 μg/dL) living near the Mbeubeuss landfill located near Dakar (Senegal). Finally, Fels et al. [73], who investigated the adverse effects of chronic low-level Pb exposure on kidney function in children from unexposed areas and from Pb-contaminated regions, showed elevated urinary excretion of two indicators of proximal tubular function, i.e., beta-2 microglobulin (β-2 M) and Clara cell protein, which positively correlated with the Pb_B_ level (133 μg/L).

Moreover, environmental Pb exposure has been reported to affect some inflammatory biomarkers. For example, Sirivarasai et al. [74] revealed significantly elevated levels of C-reactive protein (hs-CRP), one of the important biomarkers of inflammation exhibiting elevated expression during inflammatory conditions [75], in the study population with Pb_B_ of 9.21 μg/dL. Wang et al. [76] found a positive association between the plasma Pb level (8.67 μg/L) and the pro-inflammatory cytokine tumor necrosis factor alpha (TNF-α) in children. Zhang et al. [77] also demonstrated a positive association between the Pb_B_ level (37.23 μg/L) and other pro-inflammatory cytokines, such as interleukin 1 beta (IL-1β) and interleukin 6 (IL-6), in children from Guiyu, a waste dismantling center in Guangdong Province, China. In turn, Kataba et al. [78], who examined the association between chronic environmental Pb exposure and pro-inflammatory cytokines, including TNF-α, in adult males and females living in Kabwe (Zambia), showed that Pb_B_ at the level < 10 μg/dL was associated with increased TNF-α levels in female subjects.

The lipid profile, also known as a lipid panel, is one of the basic tests used to assess the body’s lipid metabolism and has been included in studies on environmental Pb exposure. For example, He et al. [79], who determined Pb levels in permanent residents aged 3–79 years in Jangxi Province (China) and Pb effects on routine hematological and biochemical indices, found that the Pb_B_ level is positively correlated with cholesterol. They showed that, for each 1 μg/L increase in Pb_B_, the risk of elevated cholesterol increases by 2.3%. A positive correlation between the levels of Pb_B_ and cholesterol was also noted by Park and Han [80], who investigated the impact of Pb_B_ levels on cardiovascular disease in adults. The same authors also observed a negative correlation between Pb_B_ levels and high-density lipoprotein (HDL). In turn, Xu et al. [81] found a positive association between the Pb_B_ level and low-density lipoprotein (LDL) in adolescents aged 12–19 years. In another study, Qiao et al. [82] investigated the impact of Pb exposure on serum metabolic profiles in preschool children. The researchers showed elevated levels of cholesterol and LDL in the serum of these children. The changes observed in the lipid profile clearly suggest that exposure to Pb affects lipid metabolism, which in turn may increase the risk of cardiovascular problems. An increase in the cholesterol and LDL levels may be a significant indicator of children’s health in the context of long-term environmental exposure to Pb.

### 1.4. Occupational Exposure to Pb—A Brief Outline

**Figure 3:**
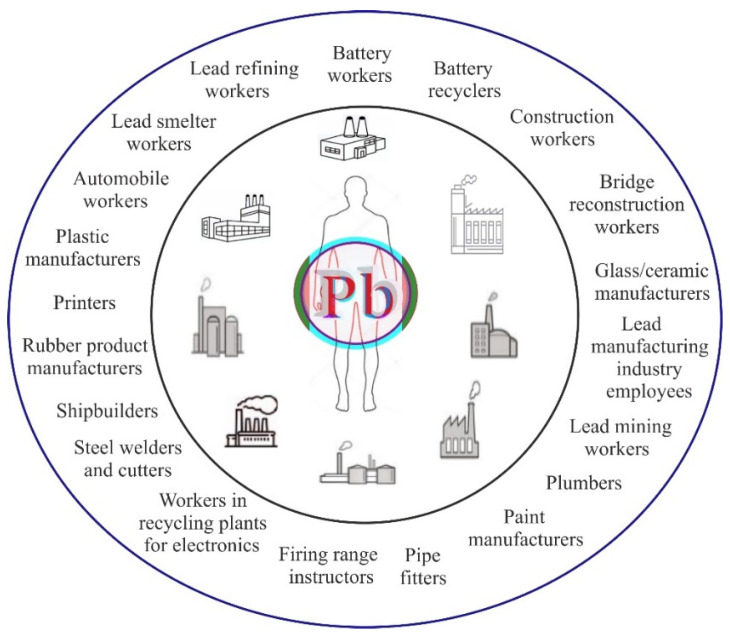
Subjects at risk of exposure to Pb in the workplace—based on the available literature cited in Section 1.4.

Occupational exposure to Pb, a widely used industrial metal that may adversely impact human health even in small amounts, occurs mainly as a result of inhalation of airborne particles [83]. Workers in numerous different sectors are exposed to this xenobiotic. Among them are battery workers and recyclers, construction workers, bridge reconstruction workers, glass and ceramic manufacturers, Pb manufacturing industry employees, Pb mining workers, plumbers, pipe fitters, firing range instructors, workers in recycling plants for electronics, steel welders and cutters, shipbuilders, rubber product manufacturers, automobile industry workers, paint manufacturers, as well as Pb smelter and refining workers [84,85,86] (Figure 3).

**Figure 4:**
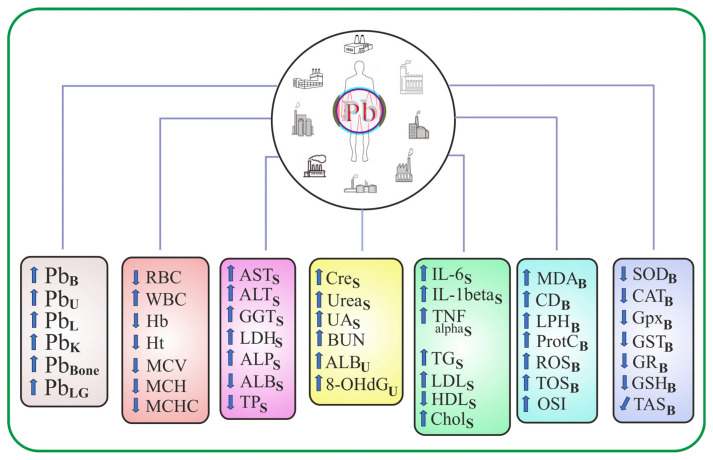
Summary of studies on unfavorable effects in hematological and biochemical indices measured in workers occupationally exposed to Pb—based on the available literature cited in Section 1.4. CAT_B_: blood catalase; CD_B_: blood conjugated dienes; Gpx_B_: blood glutathione peroxidase; GR_B_: blood glutathione reducatse; GST_B_: blood glutathione S-transferase; Pb_B_: blood lead level; LPH_B_: blood lipid hydroperoxides; MDA_B_: blood malondialdehye; ProtC_B_: blood protein carbonyl groups; ROS_B_: blood reactive oxygen species; GSH_B_: blood reduced glutathione; SOD_B_: blood superoxide dismutase; TAS_B_: blood total antioxidant status; TOS_B_: blood total oxidant status; BUN: blood urea nitrogen; Pb_Bone_: bone lead level; Ht: hematocrit; Hb: hemoglobin; Pb_K_: kidney lead level; MCH: mean corpuscular hemoglobin; MCHC: mean corpuscular hemoglobin concentration; MCV: mean corpuscular volume; Pb_L_: liver lead level; ALT_S_: serum alanine aminotransferase; ALB_S_: serum albumin; ALP_S_: serum alkaline phosphatase; AST_S_: serum aspartate aminotransferase; Chol_S_: serum cholesterol; Cre_S_: serum creatinine; GGT_S_: serum gamma-glutamyltransferase; HDL_S_: serum high density lipoprotein; IL-6_S_: serum interleukin 6; IL-1β_S_: serum interleukin 1 beta; LDH_S_: serum lactate dehydrogenase; LDL_S_: serum low-density lipoprotein; TP_S_: serum total protein; TG_S_: serum trigliceryde; TNF-alpha_S_: serum tumor necrosis factor-alpha; Urea_S_: serum urea; UA_S_: serum uric acid; OSI: oxidative stress index; Pb_LG_: pulmonal lead level; RBC: red blood cell count; WBC: white blood cell count; ALB_U_: urinary albumin; Pb_U_: urinary lead level; 8-OHdG_U:_ urinary 8-hydroksy-2′-deoxyguanosine. ↑: increase, ↓: decrease, ↙: trend towards a decrease. Gray: Pb in the blood and soft/mineralized tissues; Red: hematological indices; Pink: liver tests; Yellow: biomarkers of nephrotoxicity and DNA damage; Green: biomarkers of inflammation and lipidogram; Turquoise and blue: biomarkers of oxidative stress.

The adverse health effects of chronic occupational exposure to Pb have been widely studied. Some unfavorable alterations in hematological and biochemical parameters measured in workers occupationally exposed to this metal are summarized in Figure 4 for the reader’s convenience. Each of these indices was investigated in animal models to assess the protective effects of such antioxidants as spirulina, curcumin, garlic, vitamin C, and vitamin E (reviewed in this work) under conditions of Pb exposure. The results from these studies are collected in five tables presented in the subsequent sections of this review.

It has been noted that the occupational exposure to Pb leads to an increase in the concentration of this metal in the blood [86,87,88,89,90,91,92,93,94,95,96,97,98,99,100,101,102], urine [93,94,103], and in such internal organs as liver, kidney, bone, and lung [104]. It has also been shown that occupational exposure to Pb leads to unfavoruable changes in blood indices [89,90,93,98,105] and many biochemical parameters in the blood/urine including biomarkers of hepatotoxicity [86,87,89,97,98], biomarkers of renal function [98,100,101,106,107], biomarkers of DNA damage [108], biomarkers of inflammation [109], and biomarkers of oxidative stress [86,91,110] including those associated with the oxidation of lipids [89,92,94,95,96,97,98,103,111,112] and proteins [97]. Additionally, it has been shown that occupational exposure to Pb causes unfavorable alterations in lipid profile [86] and reduces the level/activities of non-enzymatic [91,110,113] and enzymatic antioxidants [89,91,92,94,103,111,113]. A downward trend in the total antioxidant capacity was also observed [91]. All the above-mentioned changes noted in workers occupationally exposed to Pb were detected at the specific blood Pb level summarized in Table 1.

### 1.5. Mechanisms of the Toxic Action of Pb in a Nutshell

Pb is known as a highly toxic metal with no known biological function. It can induce oxidative stress through depletion of antioxidants and enhanced formation of ROS, which are detrimental to the cell and capable of reacting with membrane lipids, especially polyunsaturated fatty acids (PUFAs), resulting in cellular damage [114]. Excessive lipid peroxidation, i.e., a complex oxidative process in which free radicals attack lipids [115], leads to alteration in the properties of membranes and membrane enzymes and further damage to proteins and nucleic acids, which is implicated in various human pathologies [116]. Pb is also capable of producing ROS by increasing the activity of xanthine oxidase [117], which catalyzes the oxidation of hypoxanthine to xanthine and then to uric acid, generating ROS [118]. Due to its high affinity for sulfhydryl (-SH) groups, Pb can also cause inhibition of thiol (-SH)-containing antioxidants such as SOD and CAT and glutathione-related enzymes, including GPx, GR, and GST [119,120]. This leads to weakening of antioxidant defense and, consequently, triggers oxidative stress, characterized by an imbalance between the production and accumulation of ROS in cells and tissues and the ability of a biological system to detoxify them [121], which, in turn, generates inflammation and cell signaling alterations in various organs and systems [15]. The above-mentioned enzymatic antioxidants neutralize the adverse impact of free radicals (SOD) [114], reduce hydrogen peroxide (H_2_O_2_) and lipid peroxides (CAT, GPx) [122], regenerate reduced glutathione by catalyzing the NADPH-dependent reduction in glutathione disulfide (GSSG) to two molecules of GSH (GR) [123], and catalyze conjugation of GSH with electrophilic substrates in response to various stress conditions to facilitate detoxification (GST) [124], thereby playing a vital role in cellular defense mechanisms. Pb also depletes GSH, which plays a key role in maintaining cellular redox homeostasis and control of ROS [125,126]. Moreover, Pb affects the activity of ALAD, a zinc-dependent enzyme, which is essential in the early stages of haem biosynthesis, as haem is the main component of Hb. The inhibition of ALAD by this toxic metal causes accumulation of delta-aminolevulinic acid (ALA) in the organism and is a crucial mechanism underlying its toxic effects, leading to the development of anemia [21,22]. Pb exhibits a high affinity for the sulphydryl groups (-SH) in the enzyme’s active site and displaces the zinc ions there, which are essential for the enzyme to function properly [127].

**Figure 5:**
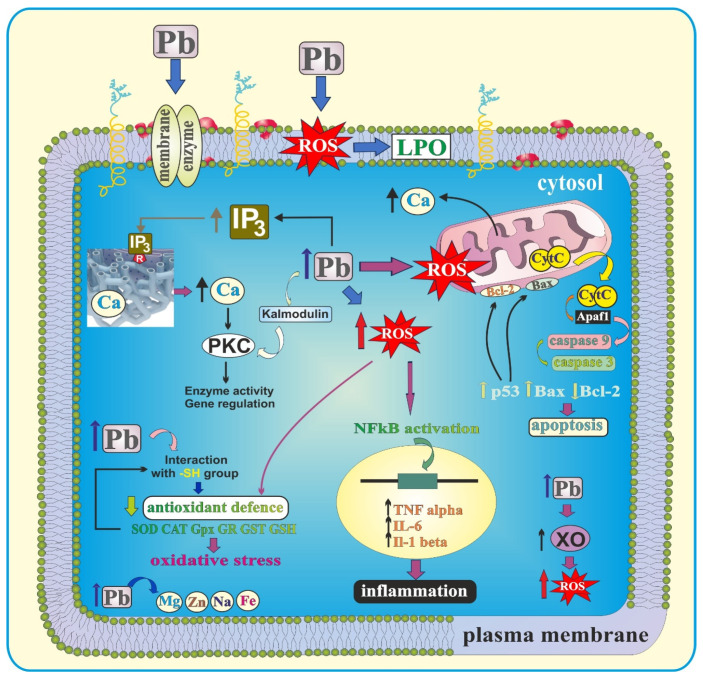
Schematic diagram summarizing the mechanisms of Pb toxicity—based on the available literature cited in Section 1.5. Apaf1: apoptotic protease activating factor 1; Bcl-2: B-cell lymphoma 2 protein; Ca: calcium; CAT: catalase; CytC: cytochrome-c; Gpx: glutathione peroxidase; GR: glutathione reductase; GST: glutathione S-transferase; IP_3_: inositol trisphosphate; IL-6: interleukin 6; IL-1beta: interleukin 1 beta; Fe: iron; Pb: lead; LPO: lipid peroxidation; Mg: magnesium; NFκB: nuclear factor kappa-light-chain-enhancer of activated B cells; Bax: pro-apoptotic protein; p53: p53 protein; PKC: protein kinase C; ROS: reactive oxygen species; R: receptor; GSH: reduced glutathione; Na: sodium; SOD: superoxide dismutase; TNF-alpha: tumor necrosis factor alpha; XO: xantine oxidase; Zn: zinc. ↑: increase; ↓: decrease.

Additionally, due to their similar biophysicochemical properties, Pb is able to compete with essential divalent cations, such as calcium (Ca^2+^), magnesium (Mg^2+^), iron (Fe^2+^), and zinc (Zn^2+^), or replace them at critical binding sites in proteins and enzymes, thereby disrupting their normal function [128]. This substitution can affect ionic transportation, signaling pathways, and cell proliferation. It has been established that a deficiency of such elements as Ca, Zn, or Fe can elevate Pb absorption [15]. Pb can also replace some monovalent ions, including sodium (Na^+^) [41], whose appropriate level is not only essential for survival but also important for hydromineral homeostasis [129]. Moreover, Pb can increase inositol trisphosphate (IP_3_) [130], which acts on receptors located in the endoplasmic reticulum (ER), leading to the release of Ca^2+^ into the cytosol. The released Ca^2+^ binds to calmodulin (Ca^2+^-binding protein), which phosphorylates protein kinase C (PKC), regulating enzyme activity and gene expression [131,132]. Pb is also capable of inducing the accumulation of ROS in the mitochondria, leading to damage to mitochondrial membranes. This metal can open the mitochondrial transition pore, stimulate the release of Ca^2+^ from mitochondria, and lead to deterioration of mitochondrial morphology and mitochondrial function [133], which may result in cell apoptosis [128] through the release of cytochrome-c (CytC) from mitochondria into the cytosol, where it interacts with apoptotic protease activating factor 1 (Apaf-1), which in turn initiates the caspase cascade executing this form of programmed cell death [134]. ROS generated by Pb exposure can also activate various inflammatory pathways, including the transcription nuclear factor kappa B (NF-κB) signaling pathway, which is involved in inflammation and stress response and regulates the expression of pro-inflammatory cytokines, such as TNF-α, IL-6, and IL-1β, thereby amplifying the inflammatory response [135]. A summary of the mechanisms of the toxic action of Pb is shown in Figure 5.

This work is an attempt to provide a thorough review of a potential antidote that may be effective in alleviating Pb toxicity, which does not perform any biological functions in the body. Bearing in mind that exposure to this toxic element remains a public health concern, we collected data from many animal-based studies to recognize whether and to what extent such antioxidants as spirulina, curcumin, and garlic, as well as vitamin C and vitamin E, are able to counteract Pb-induced toxicity. Our particular attention was focused on the effect of these antioxidants on hematological indices, biomarkers of oxidative stress, liver and kidney function, inflammation, lipid profile, apoptotic biomarkers, and histopathological changes in internal organs under conditions of Pb intoxication.

## 2. Methodology—Literature Search Strategy on the Effects of Spirulina, Curcumin, Garlic, Vitamin C, and Vitamin E Against Toxicity Induced by Pb in Animals

The literature search in English-language databases (i.e., PubMed, Scopus, and Web of Science) was conducted from September 2025 until February 2026 to identify studies on the potential benefits of spirulina, curcumin, garlic, vitamin C, and vitamin E with respect to Pb exposure. Only research articles and abstracts written in English were reviewed. Additionally, the reference lists of selected papers collected from the above-mentioned databases were manually reviewed to identify additional records (i.e., full-text papers or abstracts) that were potentially relevant to the topic. The search was focused on the ‘Title’ and ‘Abstract’ and such keywords as “lead compounds”, “lead toxicity”, “lead exposure”, “metal complexes”, “metal toxicity”, “animals”, “rats”, “mice”, “spirulina”, “curcumin”, “garlic”, “vitamin C”, “vitamin E”, “tissues”, “blood”, “urine”, “hematological indices”, “inflammantion”, “renal biomarkers”, “hepatic biomarkers”, “lipid profile” “oxidative stress”, “antioxidants”, “apoptosis” linked with “AND”.

## 3. Adverse Effects of Pb Intoxication—Animal Studies

It has been shown that intoxication with Pb causes changes in certain hematological indices [136,137,138,139,140,141,142,143,144,145,146] and biochemical parameters in the blood/urine including biomarkers of hepatotoxicity [136,138,139,140,141,142,144,145,146,147,148,149,150,151,152,153,154] and nephrotoxicity [138,139,140,142,146,155,156,157,158], biomarkers of DNA oxidation [159], pro-inflammatory cytokins [157,160,161], biomarkers of oxidative stress [138,146,149,152,160,161,162,163,164,165,166,167,168,169], indices of lipid panel [144,149,150,152,156,164,168], and biomarkers of the antioxidative system [138,143,144,146,147,149,152,159,160,162,165,167,168,169,170]. Moreover, it has been found that exposure to Pb leads to an increase in the activity of the enzyme that helps in the oxidation of hypoxanthine to xanthine and catalyzes further the oxidation of xanthine to uric acid, generating reactive oxygen species [147]. The intoxication with this metal has also been observed to increase its concentration in the blood [137,138,159,162,171,172,173] and urine [174].

**Figure 6:**
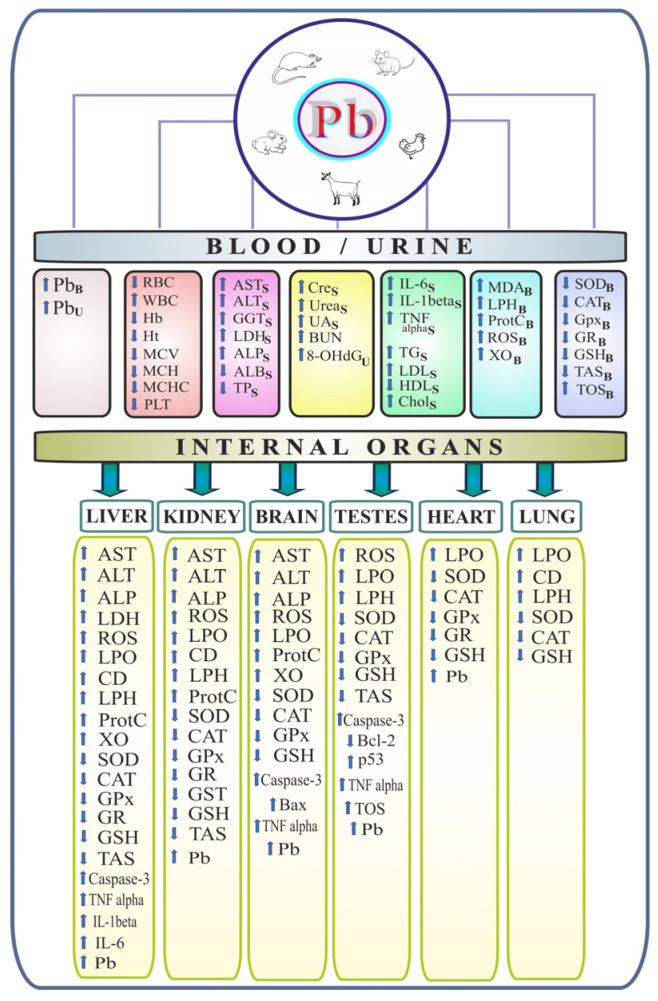
Summary of studies on unfavorable effects in hematological and biochemical indices measured in animals exposed to Pb—based on the available literature cited in Section 3. Bcl-2: B-cell lymphoma 2 protein; CAT_B_: blood catalase; Gpx_B_: blood glutathione peroxidase; GR_B_: blood glutathione reducatse; Pb_B_: blood lead level, LPH_B_: blood lipid hydroperoxides; MDA_B_: blood malondialdehye; ProtC_B_: blood protein carbonyl groups; ROS_B_: blood reactive oxygen species; GSH_B_: blood reduced glutathione; SOD_B_: blood superoxide dismutase; TAS_B_: blood total antioxidant status; TOS_B_: blood total oxidant status; BUN: blood urea nitrogen; XO_B_: blood xanthine oxidase; CD: conjugated diene; Ht: hematocrit; Hb: hemoglobin; LPO: lipid peroxidation; MCV: mean corpuscular volume; MCH: mean corpuscular hemoglobin; MCHC: mean corpuscular hemoglobin concentration; PLT: platelets count; Bax: pro-apoptotic protein; p53: p53 protein; RBC: red blood cell count; ALT_S_: serum alanine aminotransferase; ALB_S_: serum albumin; ALP_S_: serum alkaline phosphatase; AST_S_: serum aspartate aminotransferase; Chol_S_: serum cholesterol; Cre_S_: serum creatinine; GGT_S_: serum gamma-glutamyltransferase; HDL_S_: serum high density lipoprotein; IL-6_S_: serum interleukin 6; IL-1β_S_: serum interleukin 1 beta; LDH_S_: serum lactate dehydrogenase; LDL_S_: serum low-density lipoprotein; TP_S_: serum total protein; TG_S_: serum trigliceryde; TNF-α_S_: serum tumor necrosis factor-alpha; Urea_S_: serum urea; UA_S_: serum uric acid; 8-OHdG_U:_ urinary 8-hydroksy-2′-deoxyguanosine; WBC: white blood cell count. ↑: increase, ↓: decrease. Gray: Pb in the blood and urine; Red: hematological indices; Pink: liver tests; Yellow: biomarkers of nephrotoxicity and DNA damage; Green: biomarkers of inflammation and lipidogram; Turquoise and blue: biomarkers of oxidative stress.

The changes in various biochemical parameters have also been found in different internal organs of animals intoxicated with Pb. For example, it has been demonstrated that the exposure to this metal elevates the activity of such enzymes as LDH in the liver [145]; AST and ALT in the liver [175,176], kidney [137,175], and brain [137,175]; and ALP in the same organs [137,176,177]. It has also been found that intoxication with Pb elevates the ROS level in the liver [162,178], kidney [140,157,162,178], brain [178], and testes [157]; intensifies LPO in the liver [138,144,150,154,168,176,178,179,180], kidney [137,138,142,144,155,157,168,178,179,181], brain [137,153,165,178,182,183,184,185,186], heart [144,168,187,188], lung [179,187], and testes [157,185,189,190,191]; enhances the levels of CD and LPH in the liver, kidney, and lung [179] as well as the level of LPH in the testes [192]. Exposure to this xenobiotic also enhances the level of ProtC in the liver [178], kidney [140,142,178], and brain [178,186,193], and elevates the activity of XO in the liver [145] and brain [194]. Additionally, Pb has been noted to reduce the activity of other enzymes associated with oxidative stress such as SOD in the liver [141,145,150,154,168,175,176,177], kidney [137,140,142,155,157,158,168,175], brain [137,153,182,183,186,194], heart [168,187], lung [187], and testes [157,180,189,190,191,192]; CAT in the liver [150,168,176,177], kidney [137,140,142,155,157,158,168], brain [137,153,182,183,186,194], heart [157,168], lung [187], and testes [157,180,189,192]; GPx in the liver [168], kidney [140,142,155,158,168,181], brain [183,194], heart [168], and testes [191,192]; GR in the liver [168], kidney [155,168], and heart [168]; and GST in the kidney [155]. Moreover, this metal has also been found to reduce the level of GSH in the liver [143,148,150,151,154,162,168,175,176,177], kidney [137,140,148,157,168,175], brain [137,153,182,184,185,193], heart [168,187], lung [187], and testes [157,185,189]; as well as the level of TAS in the liver [138], kidney [138], and testes [195]. Biomarkers of apoptosis have been changed as well. For example, Abdrabou et al. [180] found the elevated level of expression of caspase-3 in testicular tissue. In turn, Nasr et al. [190] noted the lowered level of expression of Bcl-2 and the elevated level of expression of p53 in the same tissue. An increase in the testicular caspase-3 level was also noted by Ibrahim et al. [195]. Moreover, an increase in the caspase-3 level was found by El-Tanawy [196] and Alhusaini et al. [151] in the liver and by Galal et al. [197] in the brain. Furthermore, Han et al. [198] demonstrated the elevated level of expression of the Bax protein in the brain. Further, Abdelhamid et al. [141] showed the elevated level of such inflammatory response indices as TNF-α, IL6, and IL-1β in the liver. An increase in the level of TNF-α in the liver was also found by El-Tanawy [196]. In turn, Khalil et al. [186] and Ibrahim et al. [195] revealed the elevated level of TNF-α in the brain and testes, respectively. The latter authors also demonstrated the enhanced TOS level in the testes. The concentrations of Pb in different internal organs, such as the liver [138,145,164,171,175,176,178,199], kidney [137,138,157,164,171,175,178,181,199], brain [137,171,173,175,178,187,199], testes [157,190], bone [171,172,199], heart [187], and muscle [152,199] have been noted to be increased. The above-mentioned changes are summarized in Figure 6. In turn, unfavorable alterations noted in different organs of animals exposed to Pb are collected in Table 2.

## 4. Beneficial Effects of Spirulina, Curcumin, Garlic, Vitamin C, and Vitamin E in Pb Intoxication—Animal Studies

**Figure 7:**
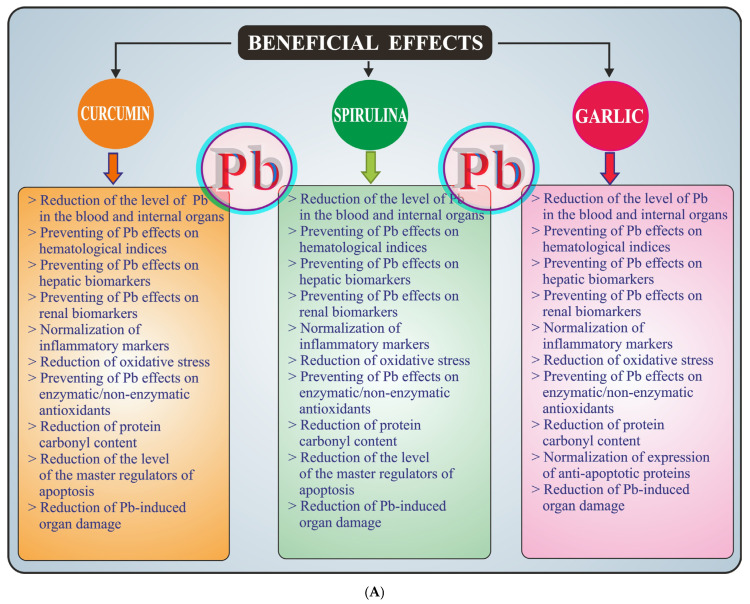

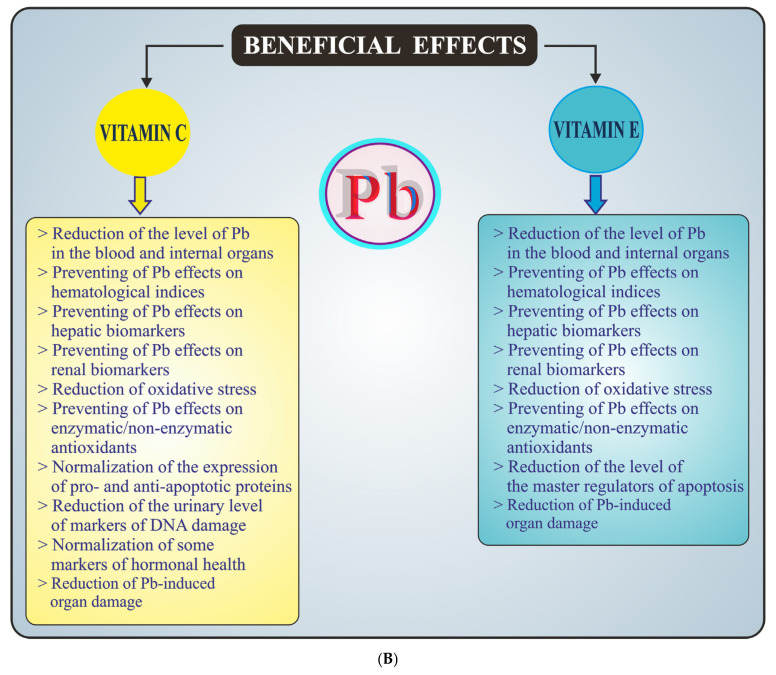
(**A**). Summary of the beneficial effects of spirulina, curcumin, and garlic in Pb poisoning—based on the results obtained from the animal-based studies reviewed in this work. The information presented in this figure derives from data provided in Table 3, Table 4 and Table 5. (**B**). Summary of the beneficial effects of vitamin C and vitamin E in Pb poisoning—based on the results obtained from the animal-based studies reviewed in this work. The information presented in this figure derives from data provided in Table 6 and Table 7.

Many studies have been conducted on animals to determine the beneficial effects of supplementation with spirulina, curcumin, garlic, vitamin C, or vitamin E under conditions of Pb exposure. Data obtained in these studies show that administration of these antioxidants during Pb poisoning leads to a reduction in Pb levels in the blood and internal organs, e.g., liver, kidney, brain, spleen, testes, heart, placenta, and bone; prevents Pb effects on hematological parameters; reverses elevated levels of hepatic, renal, and inflammatory biomarkers; and reduces the levels of ROS and biomarkers of LPO. Other beneficial effects of administration of the above-mentioned antioxidants in Pb-exposed animals were associated with the prevention of Pb effects on enzymatic and non-enzymatic biomarkers of the antioxidative system; reduction in protein oxidation; normalization of biomarkers of apoptosis; prevention of Pb-induced DNA damage; and normalization of some biomarkers of hormonal health. All these beneficial effects of spirulina, curcumin, garlic, vitamin C, and vitamin E in the conditions of Pb exposure are collected in Table 3, Table 4, Table 5, Table 6 and Table 7 and briefly summarized in Figure 7A,B.

## 5. Mechanisms of the Protective Action of Spirulina, Curcumin, Garlic, Vitamin C, and Vitamin E—A Brief Outline

Spirulina, curcumin, garlic, vitamin C, and vitamin E are important antioxidants that contribute significantly to overall health. They have been widely investigated for their diverse health benefits [259,260,261,262,263,264]. In this part of the review, the protective mechanisms of spirulina, curcumin, garlic, vitamin C, and vitamin E in the Pb-induced toxicity are summarized.

### 5.1. Microalgae—Spirulina

Spirulina (*Arthrospira platensis*), a valuable source of food with health-promoting effects, is rich in multiple bioactive compounds with potent antioxidant properties. These include phycocyanin, polysaccharides, phenolic compounds, vitamins, carotenoids, and minerals. Thanks to the presence of these components, spirulina effectively combats ROS and improves a variety of oxidative stress-induced symptoms by enhancing the body’s antioxidation capacity [259]. Kim et al. [265], who evaluated the effect of spirulina supplementation on antioxidant status and oxidative DNA damage in hypercholesterolemia-induced rabbits, showed that supplementation with this blue-green alga significantly reduced the increased LPO level in high-cholesterol-fed rabbits. Oxidative stress biomarkers such as GSH, GPx, GR, and GST were also significantly improved in the liver and red blood cells of these animals. Moreover, the level of DNA damage in lymphocytes was markedly reduced [265]. Based on these findings, it can be concluded that dietary supplementation with spirulina is a promising strategy to protect cells from LPO and oxidative DNA damage. In turn, Bermejo et al. [266], who examined the in vitro scavenger activities of different ROS, the effects on LPO, and the iron-chelating ability of *Spirulina platensis* protean extract and phycocyanin, reported that the extract inhibited the generation of hydroxyl radical (HO•) and peroxyl radical (ROO•) and suppressed the LPO process. Phycocyanin (the major component of this microalga) also inhibited the production of HO•, showing behavior similar to ion hydroxyl radical-scavenger activity. Moreover, phycocyanin inhibited the generation of ROO• and showed inhibitory activity against the LPO process [266]. Spirulina has also been reported to exhibit anti-inflammatory properties. It was demonstrated that this microalgae significantly reduces the levels of pro-inflammatory cytokines, such as TNF-α, IL-6, and IL-1β [267], and regulates interleukin 2 (IL-2), interleukin 4 (IL-4), interleukin 10 (IL-10), and interferon gamma (IFN-γ) [268]. As highlighted by some authors, the anti-inflammatory properties of spirulina may be attributable to the inhibition of cyclooxygenase-2 (COX-2) activity [269], which is known to be involved in inflammation [270,271]. It has also been reported that the up- or down-regulation of MAPK, ERK, JNK, and p38 significantly contributes to the antioxidant and anti-inflammatory activities of spirulina [268]. Phycocyanin has also been reported to reduce the levels of inflammatory factors by blocking the TLR2/NF-κB and TLR4/NF-κB pathways [272]. It can also act on the PI3K/Akt/mTOR pathway, thus directly inhibiting inflammation [272]. Several studies have also demonstrated the anti-apoptotic potential of spirulina. It has been shown that this microalga reduces cytochrome-c release from mitochondria, increases the mitochondrial membrane potential, decreases the expression of the pro-apoptotic protein Bax, increases the expression of the anti-apoptotic protein Bcl-2, suppresses the Bax/Bcl2 ratio [273], and down-regulates caspase-3 gene expression [197]. Additionally, it has metal-binding properties. It can bind metal ions from solutions and adsorb heavy metals [274,275] due to the presence of functional groups such as -OH, -C=O, -CH, and CO, which serve as active sites for metal ion binding [274]. It has been reported that spirulina can bind and excrete metals, including Pb, mercury, and cadmium, from the body [276], thereby reducing metal-induced toxicity. Additionally, *Spirulina platensis* has been demonstrated to have iron-chelating properties [266]. It affects iron ions catalyzing oxidation processes, in which highly reactive radicals, e.g., HO•, are formed from hydrogen peroxide (H_2_O_2_) [277]. The ability of spirulina to bind metals makes it a potential therapeutic agent for detoxifying heavy metals in humans.

### 5.2. Polyphenols—Curcumin

Curcumin is a lipid-soluble antioxidant of a polyphenolic nature, which exhibits a wide range of biological activities and significant pharmacological effects [260]. It exerts antioxidant activity that can interrupt the chain reactions involved in free radical generation, thereby significantly reducing oxidative stress levels [278]. This phenolic compound is able to directly neutralize ROS, as it efficiently scavenges superoxide anion (O_2_•^−^), singlet oxygen (^1^O_2_), HO•, ROO•, and H_2_O_2_. It also prevents LPO and exhibits metal chelating properties [279,280,281,282,283,284,285]. Mishra et al. [286] demonstrated that, at low superoxide concentrations, curcumin effectively causes superoxide dismutation without undergoing any chemical change. In contrast, at higher superoxide concentrations, curcumin inhibits superoxide activity by reacting with this radical species. The antioxidant potential of this bioactive polyphenol is primarily attributed to the presence of key functional moieties, including -OCH_3_, -OH, and C=C [287]. Curcumin acts as a donor of H-atoms and electrons due to its unique structure, which includes β-diketone and phenolic groups. Studies conducted by Barzegar and Moosavi-Movahedi [283] demonstrated that curcumin can undergo either electron transfer and/or H-atom donation to react with free radicals. Three different functionally dissociable groups and a β-diketone site are important factors responsible for the higher antioxidant potency of this phenolic antioxidant [283]. Moreover, curcumin has strong anti-inflammatory potential. It exerts potent anti-inflammatory effects by inhibiting the activation of the nuclear factor-κB (NF-κB) signaling pathway, regulating the mitogen-activated protein kinase extracellular signal-regulated kinase (ERK) phosphorylation cascade, and modulating the Janus kinase/signal transducer and activator of transcription (JAK/STAT) pathway [288]. In addition, curcumin can function as an anti-apoptotic agent. It regulates apoptosis-related proteins, specifically by blocking important pro-apoptotic molecules (caspase cascades), down-regulating Bax and p53 (pro-apoptotic proteins), and up-regulating Bcl-2 (anti-apoptotic protein). It enhances survival pathways through extracellular signal-regulated kinase (ERK1/2) and Bax proteins [289].

### 5.3. Spices—Garlic

Garlic (*Allium sativum*), well-known for its numerous health benefits [261], can also act as a powerful antioxidant [262]. Its antioxidant potential has been attributed to the presence of several bioactive organosulfur compounds. Among them, S-allylcysteine and S-allylmercaptocysteine have been identified as the major organosulfur components that play an important role in radical scavenging activity [290]. It has been reported that alliin (S-allyl-cysteine sulfoxide) and allicin can effectively scavenge O_2_•^−^ [291]. Moreover, alliin, S-allylcysteine, and allyl disulfide are able to scavenge HO• [292,293]. Additionally, allyl disulfide and allicin prevented LPO [292,293]. It has also been found that garlic mitigates Pb toxicity by improving antioxidant defense mechanisms and chelating ability [294]. It has also been shown to inhibit inflammatory mediators such as TNF-α and IL-1β by targeting the transcription factor nuclear factor-kappa B (NF-κB) signaling pathway. This inhibitory effect was observed in lipopolysaccharide-stimulated macrophages [295]. Garlic has also been reported to reduce apoptosis by attenuating oxidative stress, inhibiting the release of cytochrome-c from mitochondria, reducing the activity of caspase-3 (the most critical executor of apoptosis), up-regulating Bcl-2 expression, and down-regulating Bax expression [296,297].

### 5.4. Vitamins—Vitamin C and E

**Figure 8:**
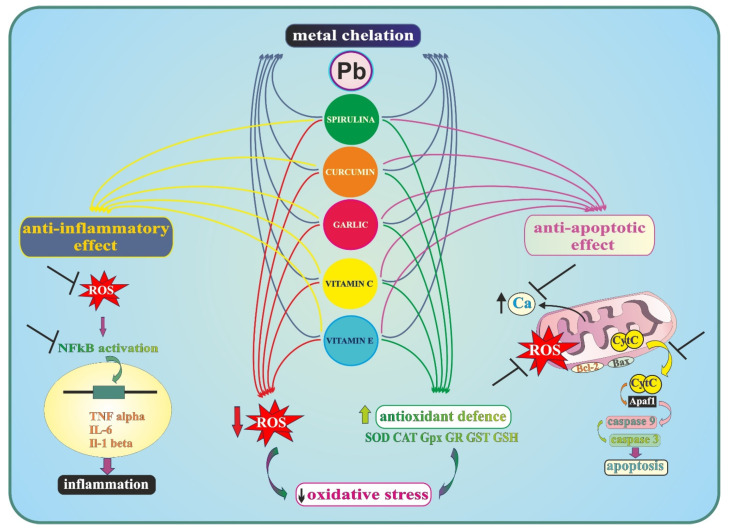
Summary of the protective mechanisms of spirulina, curcumin, garlic, vitamin C, and vitamin E against Pb toxicity—based on the available literature cited in Section 5. Apaf1: apoptotic protease activating factor 1; Bcl-2: B-cell lymphoma 2 protein; Ca: calcium; CAT: catalase; CytC: cytochrome-c; Gpx: glutathione peroxidase; GR: glutathione reductase; GST: glutathione S-transferase; IL-6: interleukin 6; IL-1beta: interleukin 1 beta; NFkB: nuclear factor kappa-light-chain-enhancer of activated B cells; Bax: pro-apoptotic protein; ROS: reactive oxygen species; GSH: reduced glutathione; SOD: superoxide dismutase; TNF-α: tumor necrosis factor alpha. ↓: decrease; ↑: increase; ⊥: inhibition; **→**: metal-binding properties; **→**: ROS scavenging activity; **→**: antioxidant effect; **→**: anti-inflammatory effect; **→**: anti-apoptotic effect.

L-ascorbic acid (vitamin C), the most abundant water-soluble compound [298], and vitamin E (α-tocopherol), a lipid-soluble substance [263], are well-known for their antioxidant properties. L-ascorbic acid is involved in the first line of antioxidant defense and can work both inside and outside cells and neutralize free radicals, thereby preventing free radical damage [264]. It is the most important antioxidant protecting extracellular fluids and the hydrophilic interior of cells [299,300]. It functions as a redox buffer and neutralizes harmful oxygen-derived species, e.g., H_2_O_2_ and ^1^O_2_ [264]. In addition, it can react with such free radicals as O_2_•^−^ and HO•, thereby participating in the initiation and propagation phases of free radical reactions. In these reactions, L-ascorbic acid is converted into the semidehydroascorbyl radical, a relatively long-lived radical, which is subsequently converted into dehydroascorbic acid. Both semidehydroascorbyl radical and dehydroascorbate are reduced back to L-ascorbic acid. This process is carried out by NADH-dependent semidehydroascorbate reductase and GSH-dependent dehydroascorbate reductase, respectively [299,300]. L-ascorbic acid, semidehydroascorbyl radical, and dehydroascorbic acid constitute a reversible oxidation-reduction system [299]. L-ascorbic acid also has the ability to protect against LPO by scavenging ROS and by one-electron reduction in lipid hydroperoxyl radicals (LOO•) via the vitamin E redox cycle [264]. It should be mentioned that L-ascorbic acid interacts with vitamin E [263] and protects this compound. The protective effect of vitamin C is associated with the regeneration of α-tocopherol, which is oxidized to tocopheryl radicals (TocO•) in reactions with free radicals. TocO• can subsequently participate in radical-radical termination reactions, giving rise to nonradical products [301]. α-Tocopherol prevents the toxic effects of O_2_•^−^ and organic radicals in the lipid environment of the cell. It can react with LOO•, which are highly reactive intermediates formed when lipid radicals react with oxygen during the propagation phase of LPO [264]. The role of vitamin E as an antioxidant against free radical-mediated LPO is its most important physiological function [263]. Vitamin C and vitamin E are also capable of chelating metal ions, thus reducing their catalytic activity to form ROS [29]. It has been demonstrated that vitamin C administered orally to Pb-exposed rats exhibited chelating properties [250]. Therefore, it may play a useful role in the removal of Pb from the organism in individuals with excessive Pb exposure. The importance of metal chelating agents should be emphasized, as they decrease redox potential and thus stabilize oxidized metal ions. Vitamin C also exerts potential effects in alleviating inflammatory status by reducing inflammatory biomarkers such as C-reactive protein (hs-CRP) and pro-inflammatory cytokines like TNF-α, IL-1β, and IL-6. It also increases the production of the anti-inflammatory cytokine interleukin 10 (IL-10) and enhances the activity of anti-inflammatory enzymes such as heme oxygenase-1 (HO-1) [302,303]. It has been reported that vitamin C provides anti-inflammatory benefits through various mechanisms and complex signaling pathways, including attenuation of the activation of key pathways such as NF-κB, MAPK, and TLR4/MyD88/PI3K [303]. Similar to vitamin C, vitamin E reduces pro-inflammatory biomarkers IL-6, IL-1β, TNF-α, and CRP, suppresses the NF-κB pathway, and reduces COX-2 activity. Moreover, both vitamins suppress apoptosis by reducing the release of cytochrome-c from mitochondria and inhibiting caspase activity [304]. A summary of the protective mechanisms of spirulina, curcumin, garlic, vitamin C, and vitamin E against Pb toxicity is presented in Figure 8.

## 6. Limitations of Using Spirulina, Curcumin, Garlic, Vitamin C, and Vitamin E—A Summarizing Note

Although spirulina, curcumin, garlic, vitamin C, and vitamin E are well recognized for their potential health benefits, largely due to their antioxidant and anti-inflammatory properties, they can cause various side effects. For example, spirulina, which has high nutritional value and is generally considered safe [305], may cause some negative effects and accumulate metals and other contaminants. A recent study conducted by Sochacka et al. [306], important in the context of consumer safety, investigated commercially available spirulina dietary supplements in Poland. The researchers demonstrated that, among the analyzed elements (Al, Ba, Cd, Co, Cr, Cu, Ga, Mo, Mn, Ni, Pb, Rb, Sr, Tl, V, Zn), such metals as aluminum (Al), manganese (Mn), strontium (Sr), and zinc (Zn) were predominant. Moreover, in the spirulina dietary supplements, which were tested in terms of the content of pharmaceutical residues, including cardiovascular drugs, antidepressants, antibiotics, and sulfonamides, caffeine was the most frequently detected compound. It was also observed that the detection patterns varied depending on the type of supplement, with higher rates of thiabendazole and metronidazole. Notably, the presence of contaminants in spirulina may originate from its cultivation. Manufacturing practices and growth environments can influence the quality and safety of spirulina supplements. When spirulina grows in uncontrolled conditions, it can accumulate heavy metals and other contaminants and thus pose a real threat to the consumer. Based on the results of their study, the authors highlighted the need for harmonized regulations, transparent labeling, and standardized monitoring protocols to ensure the safety of these products. The study conducted by Jiang and co-workers [307] also revealed the presence of cyanotoxins, including hepatotoxic microcystins produced by cyanobacterial contaminants, in 36 spirulina food products in China [307], which draws attention to the potential risks arising from exposure through the consumption of contaminated dietary supplements made from spirulina. The adverse effects of spirulina include diarrhea, nausea, bloating, upset stomach, edema, headache, and abdominal and muscle pain [308,309]. Such symptoms as headache, muscle pain, facial flushing, sweating, and difficulty concentrating have been described in individuals taking 1 g of spirulina per os daily [310]. Spirulina can also cause allergic reactions [311,312]. Spirulina has also been found to lead to rhabdomyolysis [310]. This condition was diagnosed in a 28-year-old man who was admitted to the hospital with weakness and myalgias localized in his chest and loin. He had been taking 100% Hawaiian spirulina (*A. platensis*) tablets as a dietary supplement (3 g per day) for one month. The tests have shown an elevated level of creatine kinase (CK), myoglobin, ALT, AST, LDH, and aldolase. Urinalysis revealed myoglobinuria [310]. Spirulina can also cause liver damage. Hepatotoxic effects of this microalga have been reported in a 52-year-old Japanese man who was taking spirulina. He had elevated AST and ALT levels [313]. Additionally, when administered with conventional antidiabetic drugs, spirulina can lead to a more pronounced reduction in blood glucose levels, which might increase the risk of hypoglycemia. The hypoglycemic effect of spirulina has been demonstrated in diabetic rats receiving this microalga [314]. Another study aimed to elucidate the effects of an ethanol extract and a butanol fraction of *Spirulina platensis* on insulin release and glucose homeostasis in type 2 diabetic rats showed that both the ethanol extract and the butanol fraction stimulated insulin release from mouse islets and pancreatic *β*-cells in a concentration-dependent manner. The butanol fraction also stimulated insulin secretion from perfused rat pancreas, reduced glucose absorption, and promoted gut motility [315]. Spirulina can also interact with some immunosuppressive, anticoagulant, and antiplatelet drugs [311]. Therefore, it should be used with caution when taking immunosuppressants, blood thinners, or diabetes medications. Moreover, individuals with autoimmune diseases should avoid spirulina due to its immune-boosting properties [316]. It should also be mentioned that spirulina contains phenylalanine [317]. Therefore, people with phenylketonuria, a rare metabolic disease, who cannot metabolize this amino acid, should avoid this microalgae.

Regarding curcumin, despite its well-established safety, some negative effects have also been observed. For example, in the study conducted by Lao et al. [318], some subjects receiving 500–12,000 mg of standardized curcumin powder extract experienced minimal toxicity, i.e., they developed diarrhea, headache, rash, and yellow stool. In another study, individuals receiving 0.45 g and 3.5 g curcumin daily for one to four months reported diarrhea and nausea and a rise in the serum ALP and LDH levels [319]. Moreover, curcumin has been reported to cause allergic reactions [320] and hepatotoxicity [321,322]. Unfavorable changes after curcumin administration were also noted in animals. For example, in an animal model on acute and chronic toxicities of oral curcumin-loaded nanocomplexes (CNCs), the liver function biomarkers, i.e., ALT and AST, were significantly increased in both mice and hamsters treated with high doses of CNCs (i.e., 0.8 g/kg body weight: mice, 1.61 g/kg body weight: hamsters, daily for 6 months), compared to the control group, in the chronic toxicity study. It was also demonstrated that the RBC count and Ht index were significantly increased in female mice from the high-dose CNC group. The WBC count in the female mice was also significantly higher. This increase was noted in the low-dose (0.09 g/kg body weight) and medium-dose (0.27 g/kg body weight) CNC groups. Similarly, RDW was higher in the female medium-dose and high-dose CNC groups. In turn, the MCH value was significantly lower in female mice from the medium-dose group. Moreover, animals undergoing high-dose CNC treatment exhibited slight changes in tissues, with the presence of some precipitates indicating mild inflammation [323]. It has also been reported that curcumin can induce DNA damage and chromosomal alterations in vivo at concentrations similar to those reported to exert beneficial effects [324]. These negative properties of curcumin may be mediated by its pro-oxidant potential, which may be related to the conjugated β-diketone structure of this compound [325]. In addition, curcumin was found to induce a state of overt iron deficiency anemia in mice fed with diets poor in this element. Mice fed a low-iron diet (5 mg iron/kg diet) supplemented with curcumin at 2% had a significantly reduced serum iron level, transferrin saturation, Hb level, Ht index, hepatic hepcidin content, and iron concentration in the liver and spleen. Curcumin also induced changes in liver proteins consistent with iron depletion: TfR1 turned out to be increased, and ferritin decreased [326]. These findings clearly demonstrate that curcumin affects systemic iron metabolism, particularly in conditions of subclinical iron deficiency, which may limit its use in patients with marginal or depleted iron stores [326]. Curcumin has also been revealed to inhibit the activity of drug-metabolizing enzymes, i.e., cytochrome P450 and glutathione-S-transferase [327,328]. As highlighted by some authors, the inhibition of these enzymes may result in an undesired increase in plasma concentrations of some drugs and cause toxicity in individuals supplemented with curcumin [324]. This is a well-known phytochemical that can interact with several classes of conventional drugs, including chemotherapeutic agents, antihistamines, analgesics, antidepressants, cardiovascular agents, antibiotics, immunomodulators, and anticoagulants [329].

Garlic, a polyphenolic and organosulfur-rich nutraceutical spice [330], can also cause adverse effects. These include nausea, vomiting, diarrhea, bloating, flatulence, small intestinal obstruction, epigastric and esophageal pain, tachycardia, insomnia, hematemesis, and hematochezia [329,331]. Allergic reactions have also been observed [331,332], e.g., allergic contact dermatitis, generalized urticaria, angioedema, pemphigus, anaphylaxis, and photoallergy [333]. Garlic may also alter platelet function and coagulation (with a possible risk of bleeding) and cause burns when applied to the skin [333]. Some of the side effects of garlic have also been demonstrated in rodent models. For example, the animal study conducted by Ruffin and Hunter [334], which aimed to observe possible side effects of the use of garlic as an antihypertensive agent, demonstrated that doses of garlic administered to rats at 6 h intervals daily for 28 days resulted in erratic pulse rates, abnormal electrocardiograms, weight loss, lethargy, weak soft feces, dehydration, and increased skin sensitivity on the hindlimbs and forelimbs. Another study, performed on a rat model, in which the effects of chronic garlic intake on various endogenous antioxidant enzymes and LPO in the liver and kidneys were investigated, showed that a 1000 mg/kg/day dose of garlic administered for 30 days induced marked histopathological and ultrastructural changes in both organs [335]. Changes in liver and lung tissues of rats receiving a high oral dose of garlic, i.e., 500 mg/kg/day (via gavage, daily for 4 weeks), were also reported by Alnaqeeb et al. [336]. The dose of 500 mg/kg/day has been reported to be approximately equivalent to 10 cloves/day. In another study, in which female and male rats were given 300 and 600 mg/kg per day of a garlic aqueous extract for 21 days, toxic effects affecting weight growth, biological parameters, and histological structures were found [337]. Additionally, Joseph et al. [338] observed a significant rise in AST activity and urea levels in the serum of rats fed garlic extract (2 mL/100 g body wt, intragastrically) for 10 days. An increase in serum urea, which may suggest renal function impairment, was also noted by Kamal and co-workers [339] in rats that received garlic aqueous extract orally (1 mL/rat daily) from gestational day 6 to 19. These animals had also significantly elevated serum levels of cholesterol, TG, and LDL. Moreover, fetal and maternal kidney and lung tissues exhibited a varying degree of histopathological manifestations characterized by renal necrosis, pulmonary hemorrhage, severe congestion, and vascular thickening. In turn, Chan et al. [340] examined the effect and possible modes of action of diallyl trisulfide (DAT)-rich garlic oil upon functional blood coagulation and anticoagulation factors. The results showed that administration of 50 mg garlic oil/kg body weight for 6 weeks to rats decreased RBC count, Hb level, Ht index, and PLT count, and increased WBC count and plasma fibrinogen concentration. Garlic has also been found to suppress the expression and activity of CYP2C9 in immortalized human hepatocytes (Fa2N-4 cells) [341], which clearly indicates that this popular food ingredient may reduce the serum concentrations of drugs metabolized by this enzyme. Garlic can also increase bleeding risk when combined with anticoagulant or antiplatelet medications [342,343]. Moreover, it may potentiate the blood pressure-lowering effects of certain drugs, potentially leading to hypotension [344]. Additionally, as garlic may help lower blood sugar, combining this popular cooking spice with insulin or antidiabetic agents could increase the risk of hypoglycemia [345].

Vitamin C, an essential nutrient involved in many biological functions [346], can also cause side effects. At doses of 5–10 g, it may induce nausea, diarrhea, abdominal bloating with pain, and stomach cramps [347]. Other adverse effects have also been reported. For example, Podmore et al. [348], who assessed the levels of oxidative damage to peripheral blood lymphocytes in terms of modified DNA bases in 30 healthy volunteers (16 females and 14 males aged between 17 and 49), reported that supplementation with vitamin C at the dose of 500 mg per day for 6 weeks resulted in a significant increase in the level of 8-oxoadenine, a well-known biomarker of ROS-mediated DNA damage able to induce mutagenesis [349]. Thus, ascorbic acid can act as a strong pro-oxidant in certain conditions [348]. In addition, vitamin C can serve as a source of toxic free radicals in iron- and copper-catalyzed reactions [350]. It may exert a pro-oxidant effect at high doses (1000 mg/kg body weight) [351]. Vitamin C has also been reported to increase urinary oxalate levels in a dose-dependent manner, elevating the risk of urinary stone formation [347]. Daily administration of 1 g to 2 g of ascorbic acid to both normal subjects and calcium oxalate stone-formers has been found to result in increased oxalate excretion, which plays a critical role in calcium oxalate stone formation [352]. Therefore, doses of vitamin C exceeding 1 g daily may have adverse consequences [353], and supplementation with this vitamin should be used with caution. The estimated upper limit intake of this vitamin for adults is 2 g/day [354]. It should also be mentioned that, in subjects with excessive iron accumulation, supplementation with vitamin C, which enhances the absorption of non-heme iron [355], requires special attention and careful balancing of benefits and potential risks. Vitamin C also increases the absorption of aluminum (Al) from the digestive tract [356], which can be dangerous for people with kidney diseases who take Al-containing compounds. Interaction of vitamin C with such medications, such as blood thinners, certain cancer treatments, and statins, has also been reported [357,358,359]. It is recommended to limit the intake of vitamin C in supplements to 500 mg/day [360].

Like vitamin C, vitamin E, the major lipid-soluble antioxidant essential for human health [361], can cause adverse effects as well. Transient nausea and gastric distress have been observed in some patients taking high doses of this vitamin, i.e., 2000–2500 mg/day. Diarrhea and intestinal cramps have been reported at a dose of 3200 mg/day. Non-specific side effects, which have been reported rarely, also include fatigue and muscle weakness [362]. It is estimated that the upper limit intake of this vitamin for adults is 1000 mg per day [354]. It should also have been mentioned that α-tocopherol exhibits pro-oxidant activity. Its pro-oxidant potential has been demonstrated in both in vitro [301,363] and in vivo [364] conditions. In the latter case, the authors found a 27% increase in plasma oxidation activity in patients receiving two capsules of d-α-tocopherol (250 mg; equivalent 372 IU vitamin E per capsule) daily for 6 weeks. They emphasized that this observation is important in light of a meta-analysis of the dose–response relationship between supplementation with vitamin E and total mortality. The study conducted by Miller et al. [365] demonstrated that high-dose vitamin E (i.e., ≥400 IU/day) supplementation may increase all-cause mortality. As vitamin E has pro-oxidant effects, Pearson et al. [364] suggested that the pro-oxidant properties of high-dose vitamin E may explain the increased mortality observed in adults taking such high doses (i.e., ≥400 IU/day) of this vitamin for more than one year. In light of these findings, it seems justified to review the current recommendations for the maximum vitamin E dosage of 1000 mg/day (1500 IU/day). It should also be mentioned that vitamin E may act antagonistically towards vitamin K and increase the risk of bleeding, particularly when combined with anticoagulant or antiplatelet drugs [366,367,368]. Vitamin E, due to its antioxidant potential, may also protect cancer cells from oxidative stress caused by treatment with chemotherapeutic medications [369], which could potentially reduce the effectiveness of therapy. Additionally, combining this natural antioxidant with cholesterol-lowering drugs may reduce their heart-protective effects [370].

Based on the findings presented above, it can be concluded that we are not completely free from the risk and that excessive consumption of spirulina, curcumin, garlic, vitamin C, or vitamin E may lead to unfavorable effects. Therefore, this should be taken into account when employing supplementation with these substances.

## 7. Summary and Future Research Directions

Exposure to Pb can affect all body systems, resulting in severe health problems. As intoxication with this xenobiotic remains a growing health concern worldwide, there is a high demand for developing more effective prevention and treatment strategies for Pb poisoning. The results from many animal studies included in this review clearly show that spirulina, curcumin, garlic, vitamin C, and vitamin E are able to reverse some adverse health effects of exposure to Pb, which suggests that these antioxidants can represent a natural intervention to improve health outcomes in individuals affected by exposure to this metal. However, supplements based on spirulina, curcumin, garlic, vitamin C, and vitamin E taken at inappropriate doses may have unwanted health consequences, as summarized in the previous chapter of this review. Bearing in mind their unfavorable effects, the concentrations of these antioxidants that could potentially be used during supplementation in individuals environmentally and/or occupationally exposed to Pb must be carefully considered to avoid adverse outcomes. It seems that there is a need to identify a safe dose range for these antioxidants in order to derive the benefits from their properties during Pb poisoning. To fully take advantage of their benefits, human studies with a sufficiently large sample size and a proper control group are needed to examine the long-term effects of supplementation with these antioxidants. It is important to assess their safety, efficacy, and potential risks. During longitudinal research, monitoring health outcomes will be necessary in order to assess the long-term impacts. There is also the need to conduct further animal studies to determine appropriate supplementation doses for these substances and then develop clinical trials in humans. It should also be mentioned that the quality and composition of supplements administered to individuals chronically exposed to Pb should be taken into account, as some of them, especially those based on spirulina, tend to accumulate specific metals and other contaminants [306]. In this context, it seems reasonable to monitor the concentrations of some metals in the blood of individuals chronically exposed to Pb during prolonged supplementation with such dietary supplements as spirulina. Although they can be a valuable source of nutrients offering a wide range of health benefits [371], their potential contamination may significantly influence product quality, thereby posing a health risk and affecting therapeutic efficacy.

Additionally, human clinical trials on a wide range of populations, including individuals environmentally and/or occupationally exposed to this xenobiotic and children who are vulnerable to Pb poisoning, are also vital, as they could help optimize the dosage level and time of administration. Many hematological and biochemical parameters examined in animal-based models, as presented in this review, improved after administration of spirulina, curcumin, garlic, vitamin C, or vitamin E under Pb exposure. Therefore, future trials should focus on a wide range of indices, i.e., in addition to the measurement of Pb levels in blood and urine, they could include, e.g., hematological indices, biomarkers of oxidative stress, inflammatory cytokines, liver and renal function tests, lipidogram, and hormonal panels to provide a comprehensive picture of the effects. In these studies, the administration of the above-mentioned antioxidants in combination with other natural supplements or chelating agents, which help to remove or neutralize toxic metals, should also be considered in order to identify possible interactions that may enhance therapeutic efficacy. It should not be ruled out that combined administration of these antioxidants with natural supplements or natural/synthetic chelators could ensure better protection against chronic Pb accumulation. The combined treatment could enhance antioxidant defense, improve detoxification, reduce organ damage caused by Pb intoxication, and facilitate excretion of Pb, thus offering a comprehensive approach to dealing with the toxicity of this toxic metal. Some animal-based studies showed the efficacy of combined treatment with antioxidants in conditions of Pb exposure. For example, combined treatment with vitamin C and vitamin E has been reported to enhance biological recovery from Pb-induced toxicity [372] and more effectively inhibit LPO in rats with Pb poisoning [189,373]. The combination treatment with both vitamins also more effectively enhanced the antioxidant system in Pb-intoxicated rats [189]. It has also been shown that supplementation with vitamin C may provide synergistic support to chelation therapy in managing Pb-induced oxidative stress [374]. One of the studies demonstrated that the combined administration of vitamin C with thiol chelators to Pb-exposed rats was most effective in increasing hepatic GSH levels and CAT activity [258]. Moreover, the study showed that the combined treatments with vitamin C, E, and thiol chelators were able to effectively reduce the Pb-induced decrease in the renal CAT activity and the increase in the renal LPO level. Additionally, the study demonstrated a beneficial role of vitamin E administered together with thiol chelators in reducing body Pb burden [258]. The reduced hepatic and renal Pb burden and enhanced urinary elimination of Pb in rats exposed to Pb and supplemented with vitamin C, together with thiol chelators, have also been revealed by other authors [375]. In turn, the combined administration of vitamin E with EDTA more effectively reduced the LPO levels in the liver, kidney, and brain of Pb-intoxicated rats [243]. Finally, vitamin C and curcumin administered in conjunction prevented Pb-induced brain damage [376] and exerted a protective effect against Pb-induced nephrotoxicity [377]. Thus, the studies presented above clearly show the beneficial synergistic effects of combined treatments with vitamins, vitamins with chelators, or vitamins with curcumin in the mitigation of Pb intoxication.

## 8. Conclusions

This comprehensive review shows the promising potential of spirulina, curcumin, garlic, vitamin C, and vitamin E for future applications and their potential use in novel therapies aimed at the mitigation of the toxicity of Pb, which is one of the most toxic metals to all living organisms, and for which no safe level of exposure has been established. Concurrently, it underscores the need for clinical trials to verify whether the beneficial effects observed in animal models of Pb intoxication are reproducible in humans with Pb poisoning. Such trials could help to establish the minimum effective dose with minimal side effects but maximum therapeutic benefits. Future studies are also needed to elucidate both the molecular mechanisms of action of curcumin, spirulina, garlic, vitamin C, and vitamin E against Pb toxicity, which have not yet been well recognized, and the mechanisms by which some of these antioxidants interact with chelating agents to enhance therapeutic outcomes.

## Figures and Tables

**Table 1:** Summary of the blood Pb concentrations in workers occupationally exposed to this xenobiotic, in whom side effects presented in Figure 4 were detected.

Parameter	Blood Pb Concentration	Ref.
*Pb in the blood, urine, and soft/mineralized tissues*		
Pb_B_	6.52–77.5 µg/dL, 303.33 µg/L	[86,87,88,89,90,91,92,94,95,96,97,98,99,101,102,112]
Pb_U_	17.37–30 µg/dL	[93,103]
Pb_L_	66.3 µmol/L, 19.3–105.0 µmol/L (1373.74 µg/dL)	[104]
Pb_K_	66.3 µmol/L, 19.3–105.0 µmol/L (1373.74 µg/dL)	[104]
Pb_Bone_	66.3 µmol/L, 19.3–105.0 µmol/L (1373.74 µg/dL)	[104]
Pb_Lung_	66.3 µmol/L, 19.3–105.0 µmol/L (1373.74 µg/dL)	[104]
*Hematological indices*		
RBC	30.1 µg/dL	[89]
WBC	30.1 µg/dL, ≥35 µg/dL	[89,90]
Hb	4.2–61.1 µg/dL	[89,98,105]
Ht	30.1–35 µg/dL	[89,90]
MCV	30.1–35 µg/dL	[89,90]
MCH	35 µg/dL	[90]
*Liver tests*		
AST_S_	11–30.1 µg/dL	[87,89]
ALT_S_	11–61.1 µg/dL	[86,89,98]
GGT_S_	29.1 µg/dL (9.0–61.1 µg/dL)	[98]
LDH_S_	77.5 µg/dL	[87]
ALP_S_	77.5 µg/dL	[87]
ALB_S_	29.1 µg/dL (9.0–61.1 µg/dL)	[98]
TP_S_	30–48.7 µg/dL 29.1 µg/dL (9.0–61.1 µg/dL)	[97] [98]
*Biomarkers of renal function*		
Cre_S_	>40 µg/100 mL, >60 µg/dL	[100,101,106]
Urea_S_	29.1 µg/dL (9.0–61.1 µg/dL)	[98]
UA_S_	>60 µg/dL, 9.0–61.1 µg/dL	[98,101,106]
BUN	>40 µg/100 mL, >60 µg/dL	[100,106]
ALB_U_	69 µg/100 mL	[107]
*Biomarkers of DNA oxidative damage*		
8-OHdG_U_	392 µg/L	[108]
*Biomarkers of inflammation*		
IL-6_S_	37 µg/dL	[109]
IL-1β_S_	37 µg/dL	[109]
TNF-alpha_S_	37 µg/dL	[109]
*Lipid profile*		
TG_S_	11 µg/dL	[86]
LDL_S_	11 µg/dL	[86]
HDL_S_	11 µg/dL	[86]
Chol_S_	11 µg/dL	[86]
*Biomarkers of oxidative stress*		
MDA_B_	6.52–61.1 µg/dL, 303.33 µg/L	[89,92,94,95,96,97,98,103,112]
CD_B_	30–48.7 µg/dL	[97]
LPH_B_	30–48.7 µg/dL	[97]
ProtC_B_	30–48.7 µg/dL	[97]
ROS_B_	25–35 µg/100 mL, >35 µg/100 mL	[110]
TOS_B_	11–49 µg/dL	[86,91]
OSI	49 µg/dL	[91]
*Biomarkers of antioxidative system*		
SOD_B_	30.1–57.1 µg/dL, 1.94–3.00 nmol/mL, 303.33 µg/L	[89,94,111,113]
CAT_B_	30.1–57.1 µg/dL, 303.33 µg/L	[89,94,113]
Gpx_B_	30.1–57.1 µg/dL, 303.33 µg/L	[89,91,94,113]
GST_B_	49 µg/dL	[91]
GR_B_	6.52–6.82 µg/dL	[92]
GSH_B_	25–35 µg/100 mL, >35 µg/100 mL, 49–57.1 µg/dL	[91,110,113]
TAS_B_	49 µg/dL	[91]

CAT_B_: blood catalase; CD_B_: blood conjugated dienes; Gpx_B_: blood glutathione peroxidase; GR_B_: blood glutathione reducatse; GST_B_: blood glutathione S-transferase; Pb_B_: blood lead level; LPH_B_: blood lipid hydroperoxides; MDA_B_: blood malondialdehye; ProtC_B_: blood protein carbonyl groups; ROS_B_: blood reactive oxygen species; GSH_B_: blood reduced glutathione; SOD_B_: blood superoxide dismutase; TAS_B_: blood total antioxidant status; TOS_B_: blood total oxidant status; BUN: blood urea nitrogen; Pb_Bone_: bone lead level; Ht: hematocrit; Hb: hemoglobin; Pb_K_: kidney lead level; MCH: mean corpuscular hemoglobin; MCV: mean corpuscular volume; Pb_L_: liver lead level; ALT_S_: serum alanine aminotransferase; ALB_S_: serum albumin; ALP_S_: serum alkaline phosphatase; AST_S_: serum aspartate aminotransferase; Chol_S_: serum cholesterol; Cre_S_: serum creatinine; GGT_S_: serum gamma-glutamyltransferase; HDL_S_: serum high density lipioprotein; IL-6_S_: serum interleukin 6; IL-1β_S_: serum interleukin 1 beta; LDH_S_: serum lactate dehydrogenase; LDL_S_: serum low-density lipoprotein; TP_S_: serum total protein; TG_S_: serum trigliceryde; TNF-alpha_S_: serum tumor necrosis factor-alpha; Urea_S_: serum urea; UA_S_: serum uric acid; OSI: oxidative stress index; Pb_Lung_: pulmonal lead level; RBC: red blood cell count; WBC: white blood cell count; ALB_U_: urinary albumin; Pb_U_: urinary lead level; 8-OHdG_U:_ urinary 8-hydroksy-2′-deoxyguanosine.

**Table 2:** Summary of unfavorable changes in Pb exposure in different organs—Animal Studies.

Organ	Animals	Changes	Ref.
Liver	Rodents		
		Hydropic degeneration Hepatocyte necrosis Bile duct hyperplasia Portal vein and central vein congestion	[141]
		Kuppfer cells activation Necrosis (karyolysis, eosinophilic cytoplasm, and pyknotic cells) Vacuolation Inflammatory cells infiltration	[146]
		Disruption of the normal structural organization of the hepatic lobules Loss of the characteristic cord-like arrangement of the normal liver cells Portal vein and central vein congestion Vacuolization Pyknotic nuclei Dilation of central vein and sinusoids between hepatocytes Leukocyte infiltration Fatty deposition	[177]
		Central vein congestion Dilation of sinusoids Chromatine fragmentation Pyknotic nuclei Leukocyte infiltration Precipitation of collagenous fibers Kuppfer cells activation	[200]
		Marked collagen deposition Hepatocyte degeneration	[151]
		Dilation of sinusoid lumen Fatty changes in hepatocytes Inflammation in region adjacent to blood vessels	[201]
		Periportal coagulative necrosis (pyknotic tiny nuclei, acidophilic cytoplasm, intranuclear inclusion bodies with a dense eosinophilic core) Fatty degeneration (vacuolization) Mononuclear cell infiltration of the portal triad Congestion and thrombosis of the central veins and hepatic sinusoids Dilation and congestion of central vein and hepatic sinusoids	[139]
		Severe cell damage around central vein Disruption of cytoarchitectural features Collagen deposition in the extracellular matrix	[145]
		Congestion and necrosis of liver tissue	[144]
		Impaired structure of hepatic lobules Congestion of central vein Vacuolization and necrosis of cells Dilation of central vein and hepatic sinusoids	[168]
		Thickening of liver cell plates Congestion of terminal hepatic venules and portal vein branches and sinusoidal dilation	[202]
		Loss of the characteristic cordlike arrangement Disruption of the normal structural organization of the hepatic lobules Congestion of central and portal veins Hepatocyte damage Vacuolization Dilation of central vein and hepatic sinusoids Pyknotic nuclei	[176]
		Hepatocytes necrosis (pyknotic nuclei with condensed chromatin) Congestion of portal blood vessels Perivascular edema Perivascular cuffing of round cells with scattered necrotic hepatocytes	[203]
		Aberrated liver tissue	[204]
		Increase in liver apoptotic cells	[143]
Kidney	Rodents		
		Glomerular atrophy Fragmented tufts Widening of urinary spaces Distorted proximal convoluted tubules Focal tubular necrosis Severe vacuolation Vascular changes (hypervascularization, marked dilation, congestion) Infiltration of inflammatory cells	[138]
		Tubular dilation Protein cast Necrosis (eosinophilic cytoplasm, pyknotic cells) Inflammatory cell infiltration Atrophy	[146]
		Glomerular congestion and tubular degeneration	[158]
		Kidney tubule lumen dilatation and inflammation Kidney cells degeneration	[201]
		Disorganization of the glomeruli with sloughing of the epithelium and widening of Bowman’s space Vaculation and some glomeruli shrinkage in glomerular epithelium Inflammatory infiltration in the renal medulla	[181]
		Narrowed Bowman’s spaces and fragmentation inside glomeruli Necrosis of the epithelial cells lining the tubules Inflammatory leucocytes infiltration	[140]
		Vascular congestion of epithelial cells Inflammatory leucocytes infiltration Glomeruli fragmentation Bowman’s space enlargement	[142]
		Congestion of the renal blood vessels Degenerative changes in the epithelial cells of some renal tubules Epithelial cells hypertrophy (karyo and cytomegaly) with eosinophilic intranuclear inclusion bodies Coagulative necrosis Presence of periglomerular and interstitial aggregations of round cells Thickening and hyalinization of renal arterioles Cloudy swelling and vacuolations of the convoluted tubular epithelia	[155]
		Cloudy swelling of the tubular epithelium Increased eosinophilia of the cytoplasm and indistinct cell borders Destruction and desquamation of epithelial lining in the lumen Renal casts Necrosis of the tubular epithelium (tiny and pyknotic nuclei and shrinkage of the cytoplasm) Congestion of renal blood vessels Intranuclear inclusion bodies in tubular epithelium Distension of glomeruli	[139]
		Necrotic glomeruli Presence of hyaline casts in the lumen of renal tubules	[205]
		Disruption of normal structure Vacuolar spaces and disrupted proximal and distal convoluted tubules	[168]
		Karyomegaly with eosinophilic intranuclear inclusions Glomerular damage Tubular necrosis with invading inflammatory cells	[206]
		Hyaline casts in the lumen of renal convoluted tubules Vacuolar degeneration of tubular epithelium Congestion of renal blood vessels	[203]
Spleen	Rodents		
		Distortion of spleen architecture Diffusion of white pulp into the red pulp Necrotic foci Appearance of large macrophages	[200]
		Depletion of the lymphoid follicles of the white pulps Thickening and hyalinization of blood vessels Chronic vasculitis Sub-endothelial vacuolations Splenic hemosiderosis	[139]
Brain	Rodents		
		Shrinkage and degeneration of the Purkinje and molecular layer cells with scattered glial cells Degeneration of the Purkinje cells	[207]
		Traumatic encephalopathy of the granular cell layer	[208]
		Vascular congestion	[185]
		Presence of degenerating neurons with shrunken and dark nuclei	[209]
		Cellular roundness and swelling Cellular necrosis Scattered cells with little integrity of cell membrane Appearance of dense nuclei	[210]
		Neuronal necrosis Perineuronal vacuolation and edema Dilation of Virchow-Robin spaces	[197]
		Neuronal shrinkage Presence of apoptotic cells	[198]
		Neuronal necrosis and degeneration (dark pyknotic nuclei with shrinkage of the cytoplasm) Microgliosis Neurophagia Perineuronal edema Vascular changes (subendothelial vacuolations, perivascular edema, and endothelial hypertrophy) Dilated Virchow-Robin spaces Diffuse perivascular lymphocytic infiltration	[139]
		Neuronal necrosis and degeneration (dark pyknotic nuclei with shrinkage of the cytoplasm) Multipolar cells with vacuolar spaces around them	[211]
		Reduction in Purkinje cells Presence of degenerating pyknotic cells	[212]
		Purkinje cells damage (eosinophilic cytoplasm with surrounding perineuronal vacuolation, eccentrically located nuclei, pyknotic nuclei, karyorrhexis, karyolysis)	[173]
		Diffuse edema in the cerebellar white matter (variable-sized fluid-filled spaces) Encephalomalacia (variable-sized spaces with indefinite outlines filled with cellular debris) Neurophagia Perineuronal satellite oligodendroglias surrounding small degenerated neurons with condensed chromatin and little cytoplasm Monocytic accumulations in the perivascular spaces Cerebrocortical edema	[206]
Testes	Rodents		
		Reduction in the seminiferous epithelium Necrotic debris in seminiferous tubules lumen	[141]
		Seminiferous tubules lined with degenerating spermatogenic cells with pyknotic nuclei Vacuolation and detachment of spermatogenic cells Arrested spermatogenesis Diminished number of sperm in the lumina of the seminiferous tubules	[180]
		Seminiferous tubule shrinkage Degenerative changes in the spermatogenic series Diffuse edema Decrease in Leydig cell density	[213]
		Testicular necrosis and sloughing of all layers Ischemic necrosis	[214]
		Hyperemia and edema in interstitial tissue Decrease in germinal epithelial cell number Single cell necrosis Partial necrosis in some tubules with karyorrhexis Tissue debris in some tubules Vacuolar degeneration Germinal epithelium detachment from basement membrane	[215]
		Reduction in seminiferous epithelium Empty lumen and reduction in number of luminal spermatozoa in areas	[189]
		Severe vacuolization of the testicular histoarchitecture	[185]
		Thinned irregular basement membrane in seminiferous tubules, disarrangement and damage Hyperplasia of Leydig cells	[195]
		Degeneration of spermatogenic cells and basement membranę Multinucleated giant cells within the lumina of the seminiferous tubules Impaired Leydig and Sertoli cells	[216]
		Degeneration and necrosis of the lining structures of seminiferous tubules	[190]
		Hemorrhages in the interstitial space and the lumen of the seminiferous tubules Poor spermatogenic activity	[217]
		Decreased sperm motility and motile epididymal sperm concentration	[218]
		Focal degeneration of seminiferous tubules with loss of spermatogenic series in some seminiferous tubules	[219]
		Intertubular edema of seminiferous tubules Lack of spermatocytes and spermatids in seminiferous tubules Germinal epithelium and Sertoli cells necrosis Presence of binucleated giant cells	[206]
		Testicular damage Necrosis of seminiferous tubules and loss of spermatid	[191]

**Table 3:** Summary of the beneficial effects of spirulina on Pb toxicity: an animal model.

Animals	Pb Compound	Pb Dose	Route/Time of Pb Administration	Antioxidants	Treatment Dose Route/Time of Treatment	Effects vs. Pb-Intoxicated Animals	Ref.
Wistar rats ♂	Pb acetate	100 mg/kg b.wt.	OG, 4 wk	Spirulina	OG, 0.5 mg/kg b.wt., 4 wk OG, 1 g/kg b.wt., 4 wk	↓ALT_S_, ↓AST_S_ ↓MDA_L_, ↓NO_L_ ↑SOD_L_, ↑GSH_L_ ↓TNF-α_L_, ↓caspase-3_L_	[196]
Wistar rats ♀	Pb acetate	5 mg/kg b.wt./d	dw, 5th d of gestation—14th d postpartum	Spirulina	with diet, 5% spirulina, from 5th d of gestation to 14th d of lactation	↓Pb_B_ (in pups) ↓TBARS_BR_ (in pups) Normalization of SOD_BR_, CAT_BR_, GPx_BR_ (in pups)	[183]
Wistar rats ♀	Pb acetate	343.6 mg Pb/kg b.wt./d	dw, from 5th d of gestation—14th d of postpartum	Spirulina	with diet, 5% spirulina, from 5th d of gestation to 14th d of lactation	↓Pb_B_ (in pups) ↓Pb_G_ (in pups) ↓ALT_P_, ↓AST_P_ (in pups) ↓TBARS_L_, ↓TBARS_B_ (in pups) ↓SOD_L_, ↓SOD_B_ (in pups) Normalization of CAT_L_, CAT_B_ (in pups) ↓GPx_L_ (in pups)	[220]
Wistar rats ♀	Pb acetate	343.6 mg Pb/kg b.wt./d	dw, from 5th d of gestation—14th d of lactation	Spirulina	with diet, 5% spirulina, from 5th d of gestation to 14th d of lactation	Normalization of RBC, WBC (in pups) Normalization of Hb, Ht (in pups) ↓UA_P_, ↓Urea_P_, ↓Crea_P_ (in pups) Normalization of UA_U_, Urea_U_, Crea_U_ (in pups) Normalization of BUN (in pups) Normalization of ALP_P_, GGT_P_, LDH_P_ (in pups) Normalization of LDH_K_ (in pups) ↓TBARS_K_, ↓AOPP_K_, ↓Poxid_K_, ↓H_2_O_2K_ (in pups) ↑SOD_K_, ↑CAT_K_, ↑GPx_K_, ↑GSH_K_ (in pups) Reduction in renal histopathological changes (in pups)	[140]
Wistar rats ♂	Pb acetate	25 mg/rat	i.p., 14th, 21st and 28th d of experiment	Spirulina	with diet, 5% in the standard laboratory diet, 30 d	↓ALT_P_ ↓Chol_P_, ↓TG_P_ ↓Chol_L_, ↓TG_L_ ↓MDA_L_ ↑SOD_L_, ↑CAT_L_, ↑GSH_L_ ↓MDA_K_ ↑SOD_K_, ↑CAT_K_, ↑GSH_K_	[150]
Wistar rats ♀	Pb acetate	2 g/L	dw, 30 d	Spirulina	p.o., 300 mg/kg/d, 30 d	Normalization of RBC, WBC, RET Normalization of Hb, PCV, MCHC	[221]
Albino rats ♂	Pb acetate	100 mg/kg/d	OG, 1 m	Spirulina	OG, 500 mg/kg/d, 1 m	↓MDA_T_ ↑SOD_T_, ↑CAT_T_ ↓Caspase-3_T_ ↑Testosterone_S_ Reduction in testicular histopathological and histomorphometric changes	[180]
Albino rats ♂	Pb acetate	100 mg/kg/d	OG, 1 m	Spirulina	OG, 500 mg/kg/d, 1 m	↓MDA_BR_ ↑SOD_BR_, ↑CAT_BR_ ↓Caspase-3 expression in brain Reduction in brain histopathological changes	[197]
Wistar rats ♀	Pb acetate	343.6 mg Pb/kg b.wt./d	dw, 30 d	Spirulina	in diet, 5.3 g/kg b.wt., 30 d	Normalization of RBC, WBC Normalization of Hb, PCV, MCV Normalization of MCH, MCHC ↓Crea_S_, ↓Urea_S_, ↓UA_S_ Normalization of Crea_U_, Urea_U_, UA_U_ Normalization of BUN ↑CreaC, ↓LDH_P_, ↑LDH_K_ ↓TBARS_K_ ↓AOPP_K_, ↓PC_Cont_ ↑CAT_K_, ↑SOD_K_, ↑GPx_K_, ↑GSH_K_, ↑NPSH_K_ Reduction in renal histopathological changes	[142]
Wistar rats ♂	Pb acetate	30 mg/kg b.wt.	p.o., 8 wk	Spirulina	OG, 300 mg/kg b.wt., 8 wk	↑Sperm count, ↑testosterone_S_ ↓FSH_S_, ↓LH_S_ ↓ACPT_S_, ↓LDH_S_ ↑TAC_T_, ↓TOC_T_ ↓TNF-α_T_, ↓caspase-3_T_ Reduction in testicular histopathological changes	[195]
Wistar rats ♂	Pb acetate	50 mg/kg b.wt.	i.p., 4 wk	Spirulina	OG, 300 mg/kg b.wt., 4 wk	↗RBC, ↗WBC, ↗Hb, ↗PCV ↗MCHC, ↙MCV ↗TP_S_, ↗ALB_S_, ↗T_GlobS_ ↓Crea_S_, ↓Urea_S_ ↓MDA_K_ ↑SOD_K_, ↑GSH_K_ ↓Caspase-3_K_ Reduction in renal histopathological changes	[205]
S-D rats ♂	Pb acetate	50 mg/kg b.wt.	i.p., 3x/wk, 2 wk	Spirulina	OG, 300 mg/kg b.wt., 15 d	↓Pb_B_, Pb_BR_ ↓MDA_BR_ ↑SOD_BR_, ↑CAT_BR_, ↑GSH_BR_ ↓NO_BR_, ↓PC_BR_ ↓TNF-α_BR_ ↓Caspase-3_BR_	[186]
Wistar rats ♀♂	Pb acetate	100 ppm	dw, 30 d	Spirulina	with diet, 1500 mg/kg, 30 d	↓MDA_L_, ↓MDA_K_, ↓MDA_LG_ ↓ConD_L_, ↓ConD_LG_, ↓ConD_K_ ↓HYPDX_L_, ↓HYPDX_LG_, ↓HYPDX_K_	[179]
Wistar rats ♀♂	Pb acetate	100 ppm	dw, 15 d	Spirulina	with diet, 1500 mg/kg, 15 d	↓Pb_BR_ ↓MDA_L_, ↓MDA_LG_, ↓MDA_K_, ↓MDA_C_ ↙MDA_BR_ ↑SOD_C_, ↑SOD_K_, ↑SOD_L_, ↑SOD_LG_ ↑CAT_C_, ↑CAT_K_, ↑CAT_L_, ↑CAT_LG_ ↑GSH_C_, ↑GSH_K_, ↑GSH_L_, ↑GSH_LG_	[187]
New Zealand rabbits ♂	Pb acetate	100 mg Pb/kg diet	with diet, 8 wk	Spirulina	with diet, 0.5 g/kg diet, 8 wk	↓Pb_M_ ↑Hb, ↑Ht ↗TP_S_ ↙AST_S_, ↙ALT_S_, ↓LDH_S_ ↙Chol_S_, ↙TG_S_, ↙LDL_S_ ↙UA_S_ ↑GSH_S_, ↗TAC_S_ ↗TP_S_, ↓PC_Cont_	[152]
New Zealand rabbits ♂	Pb acetate	100 mg Pb/kg diet	with diet, 8 wk	Spirulina	with diet, 1 g/kg diet, 8 wk	↓Pb_M_ ↑RBC count, ↑Hb, ↑Ht, ↙AST_S_, ↙ALT_S_, ↓LDH_S_ ↓Chol_S_, ↙TG_S_, ↙LDL_S_ ↓UA_S_ ↑TP_S_ ↓MDA_S_ ↑GSH_S_, ↗SOD_S_, ↑TAC_S_ ↓PC_Cont_ Reduction in renal/hepatic/cardiac histopathological changes	[152]
New Zealand rabbits ♂	Pb acetate	100 mg Pb/kg diet	with diet, 8 wk	Spirulina	with diet, 1.5 g/kg diet, 8 wk	↓Pb_M_ ↑RBC count, ↓WBC count, ↑Hb, ↑Ht, ↑MCHC ↓AST_S_, ↓ALT_S_, ↓LDH_S_ ↓UA_S_ ↓Chol_S_, ↓TG_S_, ↓LDL_S_ ↓MDA_S_ ↑GSH_S_, ↑SOD_S_, ↑TAC_S_ ↓PC_Cont_, ↑TP_S_ Reduction in renal/hepatic/cardiac histopathological changes	[152]

b.wt.: body weight; d: days; dw: drinking water; i.p.: intraperitoneally; OG: oral gavage; p.o.: per os; wk: weeks. CAT_B_: blood catalase; Pb_B_: blood lead level; SOD_B_: blood superoxide dismutase; TBARS_B_: blood thiobarbituric acid reactive substances; BUN: blood urea nitrogen; Caspase-3_BR_: brain caspase-3; CAT_BR_: brain catalase; GPx_BR_: brain glutathione peroxidase; Pb_BR_: brain lead level; MDA_BR_: brain malondialdehyse; NO_BR_: brain nitric oxide; PC_BR_: brain protein carbonyl level; GSH_BR_: brain reduced glutathione; SOD_BR_: brain superoxide dismutase; TBARS_BR_: brain thiobarbituric acid reactive substances; TNF-α_BR_: brain tumor necrosis factor α; CAT_C_: cardiac catalase; MDA_C_: cardiac malondialdehyde; GSH_C_: cardiac reduced glutathione; SOD_C_: cardiac superoxide dismutase; CreaC: creatinine clearance; Pb_G_: gastric lead level; Ht: hematocrit; Hb: hemoglobin; AOPP_K_: kidney advanced oxidation protein products; Caspase-3_K_: kidney caspase-3; HYPDX_K_: kidney hydroperoxide level; ConD_K_: kidney conjugated dienes; H_2_O_2K_: kidney hydrogen peroxide; CAT_K_: kidney catalase; GPx_K_: kidney glutathione peroxidase; LDH_K_: kidney lactatae dehydrogenase; MDA_K_: kidney malondialdehyde; NPSH_K_: kidney non-protein thiol level; Poxid_K_: kidney protein oxidation; GSH_K_: kidney reduced glutathione; SOD_K_: kidney superoxide dismutase; TBARS_K_: kidney thiobarbituric acid reactive substances; caspase 3_L_: liver caspase 3; CAT_L_: liver catalase; Chol_L_: liver cholesterol; ConD_L_: liver conjugated dienes; GPx_L_: liver glutathione peroxidase; HYPDX_L_: liver hydroperoxide level; MDA_L_: liver malondialdehyde; NO_L_: liver nitric oxide; GSH_L_: liver reduced glutathione; TBARS_L_: liver thiobarbituric acid reactive substances; SOD_L_: liver superoxide dismutase; TG_L_: liver triglicerydes; TNF-α_L_: liver tumor necrosis factor α; MCH: mean corpuscular hemoglobin; MCHC: mean corpuscular hemoglobin concentration; MCV: mean corpuscular volume; Pb_M:_ muscle lead level; PCV: packed cell volume; ALT_P_: plasma alanine aminotransferase; ALP_P_: plasma alkaline phosphatase; AST_P_: plasma aspartate aminotransferase; Chol_P_: plasma cholesterol; Crea_P_: plasma creatinine; GGT_P_: plasma gamma-glutamyltransferase; LDH_P_: plasma lactate dehydrogenase; TG_P_: plasma triglicerydes; Urea_P_: plasma urea; UA_P_: plasma uric acid; PC_Cont_: protein carbonyl content; ConD_LG_: pulmonal conjugated dienes; CAT_LG_: pulmonal catalase; HYPDX_LG_: pulmonal hydroperoxide level; MDA_LG_: pulmonal malondialdehyde; GSH_LG_: pulmonal reduced glutathione; SOD_LG_: pulmonal superoxide dismutase; RBC: red blood cell count; RET: reticulocyte count; ACPT_S_: serum acid phosphatase level; ALT_S_: serum alanine aminotransferase; ALB_S_: serum albumin; AST_S_: serum aspartate aminotransferase; Chol_S_: serum cholesterol; Cre_S_: serum creatinine; FSH_S_: serum follicle stimulating hormone level; LDH_S_: serum lactate dehydrogenase; LDL_S_: serum low density lipoprotein; LH_S_: serum luteinizing hormone; MDA_S_: serum malondialdehyde; GSH_S_: serum reduced glutathione; SOD_S_: serum superoxidae dismutase; Testosterone_S_: serum testosterone level; TAC_S_: serum total antioxidant capacity; T_GlobS_: serum total globulin; TP_S_: serum total protein; TG_S_: serum triglicerydes; Urea_S_: serum urea; UA_S_: serum uric acid; S-D rats: Sprague-Dawley rats; Caspase-3_T_: testicular caspase-3; CAT_T_: testicular catalase; MDA_T_: testicular malondialdehyde; SOD_T_: testicular superoxidae dismutase; TAC_T_: testicular total antioxidant capacity; TOC_T_: testicular total oxidative capacity; TNF-α_T_: testicular tumor necrosis factor α; Crea_U_: urinary creatinine; Urea_U_: urinary urea; UA_U_: urinary uric acid; WBC: white blood cell count. ↑: increase; ↗: trend towards an increase; ↓: decrease; ↙: trend towards a decrease.

**Table 4:** Summary of the beneficial effects of curcumin on Pb toxicity: an animal model.

Animals	Pb Compound	Pb Dose	Route/Time of Pb Administration	Antioxidants	Treatment Dose Route/Time of Treatment	Effects vs. Pb-Intoxicated Animals	Ref.
Albino rats ♂	Pb acetate	25 mg/kg b.wt.	i.p., daily, 7 d	Curc	i.p., 30 mg/kg b.wt., 2x/d (8 h interval), 7 d	↓Pb_B_, ↓Pb_L_, ↓Pb_K_ Normalization of RBC, WBC Normalization of Hb, Ht, EOS ↓AST_S_, ↓ALT_S_, ↓TBILI_S_ ↓Urea_S_, ↓Crea_S_ ↓MDA_S_, ↓MDA_L_, ↓MDA_K_ ↑TAC_S_, ↑TAC_L_, ↑TAC_K_ ↓Chol_S_, ↓TG_S_, ↓LDL_S_, ↑HDL_S_ Reduction in renal histopathological changes	[138]
Wistar rats ♂	Pb acetate	25 mg/kg	i.p., 3 d/wk, 8 wk	Curc	i.p., 30 mg/kg, 5 d/wk, 8 wk	↓MDA_P_, ↙MDA_H_ ↑TAC_P_	[165]
Wistar rats ♂	Pb acetate	0.1%	dw	Curc	50 mg/kg/d, NG	↓MDA_BR_ ↑GSH_BR_ Normalization of SOD_BR_, GPx_BR_ ↑AChE_BR_	[184]
Wistar rats ♂♀	Pb acetate	20 mg/kg b.wt.	i.p., 5 d	Curc	i.p., 30 mg/kg b.wt., 5 d	↑GSH_H_ ↓PC_ContH_	[193]
Albino rats ♀	Pb acetate	500 mg/L	dw, daily, 2 m	Curc	p.o., 100 mg/kg b.wt., 3x/wk, 2 m	↓Urea_S_, ↓Crea_S_ ↓MDA_K_ ↑GPx_K_, ↑GR_K_, ↑GST_K_, ↑SOD_K_ Reduction in renal histopathological changes	[155]
Wistar rats ♂	Pb acetate	50 mg/kg	p.o., daily, 45 d	Curc	p.o., 100 mg/kg, daily, 45 d	↓Pb_BR_ ↓MDA_BR_ ↑GSH_BR_ ↑SOD_BR_, ↑CAT_BR_	[182]
Wistar rats ♂	Pb acetate	50 mg/kg b.wt.	i.p., 7 d	Curc	p.o., 200 mg/kg b.wt., 4 wk 3 wk before Pb acetate in the 4th wk	↓AST_S_, ↓ALT_S_ ↓Urea_S_, ↓Crea_S_ ↑SOD_L_ mRNA, ↑CAT_L_ mRNA ↑GST_L_ mRNA, ↑GPx_L_ mRNA Reduction in hepatic/renal histopathological changes	[203]
Wistar rats ♂	Pb acetate	50 mg/kg b.wt.	OG, 1x/d on the 5th d, 1 h after Curc, 35 d	Curc	OG, 400 mg/kg b.wt., 1x/d, 40 d	↓MDA_T_ ↑SOD_T_, GPx_T_ Reduction in testicular histopathological changes	[191]
Swiss albino mice ♂	Pb acetate	25 mg/kg	i.p., 2 wk	Curc	p.o., 15 mg/kg b.wt., 2 wk	↓Pb_B_, ↓Pb_L_, ↓Pb_K_ ↓ROS_B_, ↓ROS_L_, ↓ROS_K_, ↓ROS_BR_ ↓TBARS_L_, ↓TBARS_K_, ↓TBARS_BR_ ↑GSH_L_, ↑GSH_K_, ↑GSH_BR_ ↓Prot_ContP_	[162]
Swiss albino mice	Pb acetate	25 mg/kg	i.p., 2 wk	NanoCurc	p.o., 15 mg/kg b.wt., 2 wk	↓Pb_B_, ↓Pb_L_, ↓Pb_K_, ↓Pb_BR_ ↓ROS_B_, ↓ROS_L_ ↓TBARS_L_ ↑GSH_B_ ↓GSSG_L_, ↓GSSG_K_	[162]
Wistar rats ♂	Pb acetate	20 mg/kg	i.p., 1x/d, 7 d	Curc	i.p., 30 mg/kg, 2x/d, 7 d	↓MDA_BR_ Reduction in cerebral histopathological changes	[210]
Wistar rats	Pb acetate	1000 mg/kg b.wt.	p.o., 28 d	Curc	p.o., 500 mg/kg b.wt., 28 d	↓AST_S_, ↓ALT_S_, ↓ALP_S_ ↓MDA_L_ ↑GSH_L_	[154]
S-D rats ♂	Pb acetate	25 mg/kg b.wt.	p.o., 8 wk	Curc	p.o., 30 mg/kg b.wt., 8 wk	↓Pb_B_, ↓Pb_BR_ ↓MDA_RBC_ ↑SOD_B_, ↑CAT_B_	[168]
S-D rats ♂	Pb acetate	50 mg/kg	OG, 4 wk	Curc	OG, 100 mg/kg, 4 wk OG, 200 mg/kg, 4 wk	↓Pb_BR_ ↓MDA_BR_ ↑SOD_BR_ Reduction in cerebral histopathological changes	[207]
S-D rats ♂	Pb acetate	50 mg/kg	OG, 4 wk	Curc	OG, 100 mg/kg, 4 wk OG, 200 mg/kg, 4 wk	↓Pb_L_, ↓Pb_K_ ↑Hb, ↑PCV, ↑MCV, ↑MCH, ↑MCHC ↓Crea_S_, ↓Urea_S_, ↓ALT_S_ ↓MDA_S_ ↑SOD_S_ Reduction in hepatic/renal histopathological changes	[146]
Wistar rats ♂	Pb acetate	50 mg/kg	i.p., 7 d	Curc	p.o., 200 mg/kg, 7 d	↓ALT_S_, ↓AST_S_, ↓LDH_S_ ↓MDA_L_, NO_L_ ↑SOD_L_, ↑GSH_L_ ↓TNF-α_L_ ↓Caspase-3_L_ ↑Akt, ↑GSK-3ß Reduction in hepatic histopathological changes	[151]
ICR mice ♂	Pb acetate	1% w/v	dw, 38 d	Curc	OG, 100 mg/kg b.wt., 38 d OG, 200 mg/kg b.wt., 38 d	↓MDA_RBC_, ↓MDA_P_, ↓MDA_BR_ ↑AChE_RBC_, ↑AChE_BR_, ↑AChE_P_ ↓TNF-α_P_ Improvement of cognitive processes	[161]
S-D rats ♂	Pb acetate	50 mg/kg	p.o., 4 wk	Curc-CSCaCO_3_NP Curc-CSCaCO_3_NP	p.o., 50 mg/kg, 4 wk p.o., 100 mg/kg, 4 wk	↓MDA_BR_, ↑SOD_BR_ Reduction in brain histopathological changes	[211]
Albino rats ♂	Pb acetate	100 mg/kg b.wt.	p.o., 4 wk	Curc	p.o., 400 mg/kg b.wt., 4 wk	Normalization of RBC, WBC Normalization of Hb, PCV ↓ALP_S_ ↑TP_S_, ↑ALB_S_, ↑GLOB_S_ ↓MDA_L_, ↓MDA_T_ ↑SOD_L_, CAT_L_ ↗Testosterone_S_ Reduction in hepatic/testicular histopathological changes	[141]
Wistar rats ♂	Pb acetate	30 mg/kg b.wt.	i.p., single injection	Curc CurcNPs	p.o., 60 mg/kg b.wt., 1x/d, 7 d before Pb acetate administration p.o., 30 mg/kg b.wt., 1x/d, 7 d before Pb acetate administration	↓Crea_S_, ↓UA_S_, ↓BUN ↑GSH_K_, ↑SOD_K_, ↑CAT_K_, ↑GPx_K_ Reduction in renal histopathological changes ↓Crea_S_, ↓UA_S_, ↓BUN ↑GSH_K_, ↑SOD_K_, ↑CAT_K_, ↑GPx_K_ Reduction in renal histopathological changes	[158]
Rats ♂	Pb acetate	16 mg/kg b.wt.	OG	Curc	with diet, 2000 ppm	↙MDA_S_ ↑SOD_S_, ↑CAT_S_, ↑GPx_S_ ↓IL-2_S_, ↓IL-6_S_, ↓TNF-α_S_	[160]

b.wt.: body weight; d: days; dw: drinking water; i.p.: intraperitoneally; NG: nazogastric probe; OG: oral gavage; p.o.: per os; wk: weeks. CAT_B_: blood catalase; Pb_B_: blood lead level; ROS_B_: blood reactive oxygen species; GSH_B_: blood reduced glutathione; SOD_B_: blood superoxide dismutase; BUN: blood urea nitrogen; AChE_BR_: brain acetylcholinesterase; CAT_BR_: brain catalase; GPx_BR_: brain glutathione peroxidase; Pb_BR_: brain lead level; MDA_BR_: brain malondialdehyde; GSH_BR_: brain reduced glutathione; ROS_BR_: brain reactive oxygen species; TBARS_BR_: brain thiobarbituric acid reactive substances; SOD_BR_: brain superoxide dismutase; EOS: eosynophils; AChE_RBC_: erythrocyte acetylcholinesterase; MDA_RBC_: erythrocyte malondialdehyde; GSK-3ß: glycogen synthase kinase-3 beta; Ht: hematocrit; Hb: hemoglobin; MDA_H_: hippocampal malondialdehyde; PC_ContH_: hippocampal protein carbonyl content; GSH_H_: hippocampal reduced glutathione; CAT_K_: kidney catalase; GPx_K_: kidney glutathione peroxidase; GP_K_: kidney glutathione reductase; GST_K_: kidney glutathione S-transferase; Pb_K_: kidney lead level; MDA_K_: kidney malondialdehyde; GSSG_K_: kidney oxidized glutathione; ROS_K_: kidney reactive oxygen species; GSH_K_: kidney reduced glutathione; SOD_K_: kidney superoxide dismutase; TBARS_K_: kidney thiobarbituric acid reactive substances; TAC_K_: kidney total antioxidant capacity; Caspase-3_L_: liver caspase-3; CAT_L_: liver catalase; GPx_L_: liver glutathione peroxidase; GST_L_: liver glutathione S-transferase; Pb_L_: liver lead level; MDA_L_: liver malondialdehyde; NO_L_: liver nitric oxide; GSSG_L_: liver oxidized glutathione; ROS_L_: liver reactive oxygen species; GSH_L_: liver reduced glutathione; SOD_L_: liver superoxide dismutase; TBARS_L_: liver thiobarbituric acid reactive substances; TAC_L_: liver total antioxidant capacity; TNF-α_L_: liver tumor necrosis factor α; MCV: mean corpuscular volume; MCH: mean corpuscular hemoglobin; MCHC: mean corpuscular hemoglobin concentration; PCV: packed cell volume; AChE_P_: plasma acetylcholinesterase; MDA_P_: plasma malondialdehyde; Prot_ContP:_ plasma protein content; TAC_P_: plasma total antioxidant capacity; TNF-α_P_: plasma tumor necrosis factor α; Akt: protein kinase B; RBC: red blood cell count; ALT_S_: serum alanine aminotransferase; ALB_S_: serum albumin; ALP_P_: serum alkaline phosphatase; AST_S_: serum aspartate aminotransferase; CAT_S_: serum catalase; Chol_S_: serum cholesterol; Crea_S_: serum creatinine; GLOB_S_: serum globulin; GPx_S_: serum glutathione peroxidase; HDL_S_: serum high density lipoprotein; IL-2_S_: serum interleukin 2; IL-6_S_: serum interleukin 6; LDH_S_: serum lactate dehydrogenase; LDL_S_: serum low density lipoprotein; MDA_S_: serum malondialdehyde; SOD_S_: serum superoxide dismutase; Testosterone_S_: serum testosterone; TAC_S_: serum total antioxidant capacity; TBILI_S_: serum total bilirubin; TP_S_: serum total protein; TG_S_: serum triglicerydes; TNF-α_S_: serum tumor necrosis factor α; Urea_S_: serum urea; UA_S_: serum uric acid; S-D rats: Sprague-Dawley rats; GPx_T_: testicular glutathione peroxidase; MDA_T_: testicular malondialdehyde; SOD_T_: testicular superoxide dismutase; WBC: white blood cell count. ↑: increase; ↗: trend towards an increase; ↓: decrease; ↙: trend towards a decrease.

**Table 5:** Summary of the beneficial effects of garlic on Pb toxicity: an animal model.

Animals	Pb Compound	Pb Dose	Route/Time of Pb Administration	Antioxidants	Treatment Dose Route/Time of Treatment	Effects vs. Pb-Intoxicated Animals	Ref.
Wistar rats ♂	Pb acetate	1000 ppm	OG, 35 d	Garlic	OG, 400 mg/kg, 35 d	↗SOD_T_ ↑Sperm viability	[222]
Wistar rats ♂	Pb acetate	1000 ppm	dw, 28 d	Garlic	OG, 500 mg/kg/day, 28 d	↑Sperm viability Reduction in testicular histopathological changes	[215]
Swiss albino mice	Pb acetate	100 mg/kg b.wt.	p.o., 42 d	Garlic	p.o., 1 mL/mice b.wt., 42 d	↓Pb_B_, ↓Pb_L_, ↓Pb_K_, ↓Pb_BR_, ↓Pb_F_ Normalization of RBC, WBC Normalization of Hb, PCV ↙AST_S_	[136]
Albino rats ♂	Pb acetate	15 mg/kg b.wt.	i.p., 1x/d, 7 d	Garlic	p.o., 50 mg/kg b.wt., 1 h before Pb acetate, 7 d	Normalization of Hb ↓ALT_S_, ↓ALP_S_ Normalization of SOD_L_, CAT_L_ ↓LPO_L_, ↓XO_L_, ↓LDH_L_ ↑GSH_L_ Reduction in hepatic histopathological changes	[145]
Charles Forster albino rats ♂	Pb acetate	15 mg/kg b.wt.	i.p., 7 d	Garlic	p.o., 50 mg/kg b.wt., 1 h before Pb acetate, 7 d	↓AST_S_, ↓LDH_S_, ↓LPO_C_, ↓XO_C_ Normalization of GSH_C_ Normalization of Cu/Zn-SOD_C_ and CAT_C_ Normalization of ICDH_C_, αKGDH_C_, SCDH_C_ Normalization of Col_ContC_ Prevention of cardiac fibrosis Reduction in cardiac histopathological changes	[147]
Wistar rats ♂	Pb acetate	1000 mg/L	dw, 2 wk	Garlic	p.o., 250 mg/kg b.wt./d, 2 wk p.o., 500 mg/kg b.wt./d, 2 wk	↓ROS_BR_, ↓ROS_L_, ↓ROS_K_ ↓LPP_BR_, ↓LPP_L_, ↓LPP_K_ ↓TPC_ContBR_, ↓TPC_ContL_, ↓TPC_ContK_	[178]
BALB/c mice ♂♀	Pb acetate	1 ppm	dw, 28 d	Garlic	dw, 100 mg/mL dw, 50 mg/mL dw, 25 mg/mL dw, 12.5 mg/mL from 29th d—50th d after Pb exposure	↓Pb_L_, ↓Pb_K_, ↓Pb_C_, ↓Pb_SP_, ↓Pb_B_ ↓Pb_L_, ↓Pb_K_, ↓Pb_C_, ↓Pb_SP_, ↓Pb_B_ ↓Pb_L_, ↓Pb_K_, ↓Pb_C_, ↓Pb_SP_, ↓Pb_B_ ↓Pb_L_, ↓Pb_K_, ↓Pb_C_, ↓Pb_SP_, ↓Pb_B_	[223]
S-D rats ♀	Pb acetate	160 mg/L	dw, 1 m	Garlic	OG, 100 mg/kg/d, 1 m	↓Pb_B_ Normalization of Hb ↓ALT_S_, ↓AST_S_ Normalization of CAT_S_ ↓TBARS_C_, ↓TBARS_K_, ↓TBARS_L_, ↓TBARS_S_ ↑TP_S_, ↑TP_L_, ↑TP_C_ ↓Chol_S_, ↓TG_S_, ↓LDL_S_, ↑HDL_S_ ↓Chol_C_, ↓TG_C_, ↓LDL_C_, ↑HDL_C_ ↓Chol_L_, ↓TG_L_, ↓LDL_L_, ↑HDL_L_ Reduction in hepatic histopathological changes	[144]
S-D albino rats ♂	Pb acetate	10 mg/kg/d	dw, 1 m	Garlic	p.o., 100 mg/kg b.wt., 1 m	↓Pb_S_ Normalization of Hb, RBC, WBC ↓Chol_S_, ↓Chol_L_, ↓Chol_C_, ↓Chol_K_ ↓LDL_S_, ↑HDL_S_ ↓TG_S_, ↓TG_L_, ↓TG_C_, ↓TG_K_ ↓TBARS_C_, ↓TBARS_K_, ↓TBARS_L_, ↓TBARS_S_ ↑GSH_C_, ↑GSH_K_, ↑GSH_L_, ↑GSH_S_ ↑CAT_C_, ↑CAT_K_, ↑CAT_L_, ↑CAT_S_ ↑SOD_C_, ↑SOD_K_, ↑SOD_L_, ↑SOD_S_ ↑GR_C_, ↑GR_K_, ↑GR_L_, ↑GR_S_ ↑GPx_C_, ↑GPx_K_, ↑GPx_L_, ↑GPx_S_ Reduction in hepatic/renal histopathological changes	[168]
Albino rats ♂	Pb acetate	8 mg/kg b.wt./d	i.p., 10 wk	Garlic	OG, 200 mg/kg b.wt., 10 wk	↓Pb_K_, ↓Pb_T_ ↓Cre_S_, ↓Urea_S_, ↓UA_S_ ↓MDA_K_, ↓MDA_T_ ↑SOD_K_, ↑SOD_T_ ↑CAT_K_, ↑CAT_T_ ↑GSH_K_, ↑GSH_T_ ↓TNF-α_S_ ↑Testosterone_S_ ↑Sperm concentration ↑Sperm motility ↓Percentage of dead sperms ↓Rate of abnormal sperms	[157]
Albino rats ♂	Pb acetate	100 mg/kg/d	OG, 1 m	Garlic	OG, 600 mg/kg/d, 1 m	↓MDA_T_ ↑SOD_T_, ↑CAT_T_ ↓Caspase-3_T_ ↑Testosterone_S_ Reduction in testicular histopathological and histomorphometric changes	[180]
Wistar rats ♀	Pb acetate	1500 ppm	dw, 0–21 d of pregnancy	Garlic	OG, 400 mg/kg, 0–21 d of pregnancy	↓Pb_B_ in neonates ↓Pb_Bone_ in neonates Normalization of bone ossification in neonates	[172]
Albino rats ♂	Pb acetate	20 mg/kg/d	p.o., 6 d/wk, 3 m	Garlic Sylimarin	p.o., 20 mg/kg/d, 1 m p.o., 1000 mg/kg/d, 1 m	↓Pb_K_ ↓MDA_K_ ↑GPx_K_ Reduction in renal histopathological changes	[181]
Albino rats ♂	Pb acetate	100 mg/kg/d	OG, 1 m	Garlic	OG, 600 mg/kg/d, 1 m	↓MDA_BR_ ↑SOD_BR_, ↑CAT_BR_ ↓Caspase-3 expression in brain Reduction in brain histopathological changes	[197]
Albino rats ♀	Pb acetate	20 mg/kg b.wt.	p.o., daily, 10 wk	Garlic	p.o., 1 mg/rat, daily, 10 wk	Normalization of RBC, Hb, PCV, MCV ↓AST_S_, ↓ALT_S_ ↓Crea_S_ Reduction in hepatic/renal/cerebral/splenic histopathological changes	[139]
Albino rats ♂	Pb acetate	50 mg/L	dw, 42 d	Garlic	OG, 200 mg/kg b.wt./d, 42 d	↓Pb_T_ ↓MDA_T_ ↑SOD_T_ ↓p53_T_ expression ↑Bcl-2_T_ expression ↓Caspase-3_T_, Bax_T_ expression Reduction in sperm abnormalities Improvement of seminal picture Reduction in testicular histopathological changes	[190]
S-D albino rats ♀	Pb acetate	160 mg/kg b.wt. 320 mg/kg b.wt.	OG, 1–20 d of pregnancy	Garlic	OG, 250 mg/kg b.wt./d 1–20 d of pregnancy	↓Pb_PL_ ↓Maternal Pb_B_, ↓Maternal Pb_BR_ ↓Fetal Pb_BR_ ↑Fetal weight Reduction in cerebral histopathological changes	[173]
Rats ♀	Pb acetate	5 mg/kg b.wt./d	p.o., 6 wk	Garlic	p.o., 100 mg/kg b.wt., 6 wk p.o., 200 mg/kg b.wt., 6 wk p.o., 400 mg/kg b.wt., 6 wk	↓Pb_B_, ↓Pb_L_, ↓Pb_K_ ↓Pb_B_, ↓Pb_L_, ↓Pb_K_, ↓Pb_BR_, ↓Pb_Bone_ ↓Pb_B_, ↓Pb_L_, ↓Pb_K_, ↓Pb_BR_, ↓Pb_Bone_	[171]
Swiss albino mice ♂	Pb nitrate	50 mg/kg b.wt./d	OG, 40 d	Garlic	OG, 250 mg/kg b.wt./d, 30 d OG, 500 mg/kg b.wt./d, 30 d	↓Pb_B_, ↓Pb_K_, ↓Pb_BR_ Normalization of RBC, WBC Normalization of Hb ↓MDA_K_, ↑CAT_K_, ↑SOD_K_, ↑GSH_K_ ↓MDA_BR_, ↑CAT_BR_, ↑SOD_BR_, ↑GSH_BR_ ↓AST_K_, ↓ALT_K_, ↓ALP_K_ ↓AST_BR_, ↓ALT_BR_, ↓ALP_BR_ ↑TP_K_, ↓Chol_K_ ↑TP_BR_, ↓Chol_BR_	[137]
Swiss albino mice ♂	Pb nitrate	50 mg/kg b.wt./d	OG, 40 d	Garlic	OG, 100 mg/kg b.wt./d, 30 d OG, 250 mg/kg b.wt./d, 30 d	↓Pb_B_, ↓Pb_K_, ↓Pb_BR_ Normalization of RBC, WBC Normalization of Hb ↓MDA_K_, ↑CAT_K_, ↑SOD_K_, ↑GSH_K_ ↓MDA_BR_, ↑CAT_BR_, ↑GSH_BR_ ↓AST_K_, ↓ALT_K_, ↓ALP_K_ ↓AST_BR_, ↓ALT_BR_, ↓ALP_BR_ ↑TP_K_, ↓Chol_K_ ↑TP_BR_, ↓Chol_BR_	[137]
Swiss albino mice ♂	Pb nitrate	50 mg/kg b.wt./d	OG, 40 d	Garlic	OG, 250 mg/kg b.wt./d, 30 d OG, 500 mg/kg b.wt./d, 30 d	↓Pb_L_ ↓LPO_L_ ↑CAT_L_, ↑SOD_L_, ↑GSH_L_ ↓AST_L_, ↓ALT_L_, ↓ACPT_L_, ↓ALP_L_ ↑TP_L_, ↓Chol_L_ Reduction in hepatic histopathological changes	[176]
Swiss albino mice ♂	Pb nitrate	50 mg/kg b.wt./d	OG, 40 d	Garlic	OG, 100 mg/kg b.wt./d, 30 d OG, 250 mg/kg b.wt./d, 30 d	↓Pb_L_ ↓LPO_L_ ↑CAT_L_, ↑GSH_L_ ↓AST_L_, ↓ALT_L_, ↓ACPT_L_, ↓ALP_L_ ↑TP_L_, ↓Chol_L_ Reduction in hepatic histopathological changes	[176]
Wistar Rats ♀	Pb acetate	100 µmol/kg b.wt.	i.p., 7 d	Garlic	200 g minced garlic/kg diet, 7 d	↓ALT_S_, ↓ALP_S_	[224]
Rats ♀	Pb acetate	100 µmol/kg b.wt.	i.p., 7 d	Garlic	200 g minced garlic/kg diet, 7 d	↓Pb_BR_, ↓Pb_L_, ↓Pb_K_	[199]
Albino rats ♂	Pb acetate	5 mg/kg/4 mL	i.p., 1x/d, 6 wk	Garlic	OG, 40 mg/kg/4 mL, 2x/d, 6 wk	↓Pb_B_, ↓Pb_K_, ↓Pb_L_ ↓ALT_S_, ↓AST_S_ ↑GSH_K_, ↑GSH_L_	[148]
Wistar rats ♂	Pb nitrate	2 mg/kg b.wt.	p.o., 6 wk	Garlic	p.o., 300 mg/kg b.wt., 6 wk	↑RtTSPR, ↑LtTSPR ↑RtESPRs, ↑LtESPRs ↑Spermatogenic cells Reduction in testicular histopathological changes	[217]
BALB/c mice ♀	Pb acetate	30 mg/kg/d	OG, 2 m	Garlic	OG, 500 mg/kg/day, 2 m	↗PFC Normalization of the ovarian morphology	[225]
Mice ♂	Pb acetate	5 mg/kg/d	p.o., 4 wk after garlic/garlic tablet administration	Garlic Garlic Garlic Garlic tablet Garlic tablet Garlic tablet	p.o., 125 mg/kg/d, 4 wk p.o., 250 mg/kg/d, 4 wk p.o., 500 mg/kg/d, 4 wk p.o., 1/4 tablet, 4 wk p.o., 1/8 tablet, 4 wk p.o., 1/16 tablet, 4 wk	↓Pb_B_, ↓Pb_Bone_, ↓Pb_K_, ↓Pb_L_ ↓Pb_B_, ↓Pb_Bone_, ↓Pb_K_, ↓Pb_L_ ↓Pb_B_, ↓Pb_Bone_, ↓Pb_K_, ↓Pb_L_ ↓Pb_B_, ↓Pb_Bone_, ↓Pb_K_, ↓Pb_L_ ↓Pb_B_, ↓Pb_Bone_, ↓Pb_K_, ↓Pb_L_ ↓Pb_B_, ↓Pb_Bone_, ↓Pb_K_, ↓Pb_L_	[226]
BALB/c mice ♀	Pb acetate	15 mg Pb/kg b.wt.	OG, 15 d	Garlic	p.o., 750 mg/kg b.wt., 10 d after Pb acetate exposure	↓AST_S_, ↓ALT_S_ ↓Crea_S_, ↓Urea_S_ ↓MDA_RBC_ ↓H_2_O_2RBC_ ↓SOD_RBC_, ↑CAT_RBC_ ↑GSH/GSSG_RBC_ ↑T-SH_ContRBC_	[163]
Swiss albino mice ♂	Pb nitrate	2 mg/kg b.wt.	i.p., 25 d	Garlic	p.o., 250 mg/kg b.wt., 25 d	↓Pb_L_ ↑GSH_L_, ↑GSH_K_ ↓MDA_K_ ↓ALT_K_, ↓AST_BR_, ↓ALT_BR_ ↑TP_L_, ↓Chol_L_, ↓Chol_K_	[175]
Swiss albino mice ♂	Pb nitrate	2 mg/kg b.wt.	i.p., 25 d	Garlic	p.o., 500 mg/kg b.wt., 25 d	↓Pb_BR_, ↓Pb_K_, ↓Pb_L_ ↓AST_L_, ↓AST_BR_, ↓ALT_L_, ↓ALT_K_, ↓ALT_BR_ ↓MDA_L_, ↓MDA_K_, ↓MDA_BR_ ↑SOD_L_, ↑SOD_BR_ ↑GSH_L_, ↑GSH_K_ ↑TP_L_, ↑TP_BR_ ↓Chol_L_, ↓Chol_K_	[175]
Albino rats ♂	Pb acetate	2.5 mg/kg	OG, 15 d	Garlic	100 mg/kg, OG, 12 h after Pb acetate administration, 15 d	↓Chromosomal aberrations Reduction in hepatic histopathological changes	[204]
Wistar rats ♂	Pb acetate	2.5 mg/kg	OG, 4 wk	Garlic	OG, 100 mg/kg, 4 wk	↓Chromosomal aberrations ↓TBARS_L_	[227]
Wistar rats ♂	Pb acetate	15 mg/kg/d	i.p., 10 d	Garlic	p.o., 50 mg/kg/d, 28 d	↑Sperm count ↑Sperm motility and viability ↑GSH_BR_, ↑GSH_T_ ↓TBARS_BR_, ↓TBARS_T_ ↑FSH_P_, ↑LH_P_, ↑Testosterone_P_ Reduction in pituitary and testicular histopathological	[185]
Wistar rats ♂	Pb acetate	15 mg/kg/d	i.p., 10 d	Garlic	p.o., 100 mg/kg/d, 28 d	↑Sperm count ↑Sperm motility and viability ↑GSH_BR_, ↑GSH_T_ ↓TBARS_BR_, ↓TBARS_T_ ↑FSH_P_, ↑LH_P_, ↑Testosterone_P_ Reduction in pituitary and testicular histopathological changes	[185]
Wistar rats ♂	Pb acetate	15 mg/kg/d	i.p., 10 d	Garlic	p.o., 200 mg/kg/d, 28 d	↑Sperm count ↑Sperm motility and viability ↑GSH_BR_, ↑GSH_T_ ↓TBARS_BR_, ↓TBARS_T_ ↑FSH_P_, ↑LH_P_, ↑Testosterone_P_ Reduction in pituitary and testicular histopathological changes	[185]
Wistar rats ♂	Pb acetate	120 mg/kg b.wt.	p.o., from 15th to 21st d of experiment	Garlic	p.o., 300 mg/kg b.wt., from the 1st to 21st d of experiment	↗SOD_B_ ↙MDA_B_ Reduction in cerebral histopathological changes	[208]
Wistar rats ♂	Pb acetate	120 mg/kg b.wt.	p.o., from 15th to 21st d of experiment	Garlic	p.o., 500 mg/kg b.wt., from the 1st to 21st d of experiment	↗SOD_B_ ↙MDA_B_ Reduction in cerebral histopathological changes	[208]
Chickens	Pb acetate	5 mg Pb/kg b.wt.	n/a	Garlic	n/a	↓Pb_L_, ↓Pb_M_	[228]
Broiler chickens	Pb acetate	100 mg/kg b.wt.	with diet, 42 d	Garlic	with diet, 1% garlic, 42 d	↓Pb_Bone_, ↓Pb_BR_, ↓Pb_K_, ↓Pb_L_, ↓Pb_M_, ↓Pb_SP_, ↓Pb_B_	[229]
Broiler chickens	Pb acetate	100 mg/kg b.wt.	with diet, 42 d	Garlic	with diet, 2% garlic, 42 d	↓Pb_Bone_, ↓Pb_BR_, ↓Pb_K_, ↓Pb_L_, ↓Pb_M_, ↓Pb_SP_, ↓Pb_B_	[229]
Broiler chickens	Pb acetate	100 mg/kg b.wt.	with diet, 42 d	Garlic	with diet, 4% garlic, 42 d	↓Pb_Bone_, ↓Pb_BR_, ↓Pb_K_, ↓Pb_L_, ↓Pb_M_, ↓Pb_SP_, ↓Pb_B_	[229]
Hubbard Classic broiler chickens ♂♀	Pb acetate	100 mg/kg b.wt.	with diet, 42 d	Garlic	with diet, 1% garlic, 42 d	↓UA_S_, ↓Crea_S_, ↙BUN ↙Chol_B_, ↓TG_B_, ↓LDL_B_, ↑HDL_B_	[156]
Hubbard Classic broiler chickens ♂♀	Pb acetate	100 mg/kg b.wt.	with diet, 42 d	Garlic	with diet, 2% garlic, 42 d	↓UA_S_, ↓Crea_S_, ↓BUN ↓Chol_B_, ↓TG_B_, ↓LDL_B_, ↑HDL_B_	[156]
Hubbard Classic broiler chickens ♂♀	Pb acetate	100 mg/kg b.wt.	with diet, 42 d	Garlic	with diet, 4% garlic, 42 d	↓UA_S_, ↓Crea_S_, ↓BUN, ↙Chol_B_, ↓TG_B_, ↓LDL_B_, ↑HDL_B_	[156]
Rabbits ♂	Pb acetate	40 mg/kg b.wt./d	OG, 20 d	Garlic	OG, 0.5 mg/kg b.wt./d, 20 d	↓Pb_B_ ↑AChE_BR_ fore brain region ↑AChE_BR_ mid brain region ↑AChE_BR_ hind brain region ↑AChE_SC_, ↑AChE_S_, ↑BChE_S_	[230]
New Zealand rabbits ♂	Pb acetate	15 mg/kg b.wt.	p.o., 5 d/wk, 2 m	Garlic	p.o., 400 mg/kg b.wt., 5 d/wk 2 m, 1 h before Pb acetate administration	Reduction in lung alveoli histopathological changes	[231]
Goats ♀	Pb acetate	80 mg/kg b.wt.	p.o., 1x/d, 5 d	Garlic	p.o., 45 g/d/animal, 5 d	↓Pb_Bone_, ↓Pb_C_, ↓Pb_K_ ↓Pb_L_, ↓Pb_M_, ↓Pb_Lung_	[232]
Goats ♀	Pb acetate	80 mg/kg b.wt.	p.o., 5 d	Garlic	p.o., 45 g/animal/day, 5 d	↓Pb_Bone_, ↓Pb_C_, ↓Pb_K_ ↓Pb_L_, ↓Pb_M_, ↓Pb_Lung_, ↓Pb_S_ ↑Pb_U_	[174]
New Zealand White rabbits ♂♀	Pb acetate	0.05%	with diet	Garlic	with diet, 0.8%	↑TP_S_, ↑ALB_S_, ↑Glob_S_ ↓AST_S_, ↓ALT_S_ ↓Chol_S_, ↓TG_S_ ↓Crea_S_, ↓Urea_S_ ↓MDA_L_ ↑GSH_L_, ↑GST_L_, ↑GP_XL_	[233]
Swiss albino mice ♂	Pb nitrate	50 mg/kg b.wt.	p.o., 30 d	Garlic	p.o., 80 mg/kg, from the 12th d of the experiment	↑SOD_LG_, ↑CAT_LG_, ↑GPx_LG_, ↑GSH_LG_ ↑TPC_ContLG_, ↓Chol_LG_ ↓LDH_LG_ Reduction in pulmonary histopathological changes	[234]
Swiss albino mice ♂	Pb nitrate	50 mg/kg b.wt.	OG, 30 d	Garlic	OG, 50 mg/kg b.wt., from the 12th d of the experiment	↙ALT_LG_ ↓AST_LG_	[235]
Swiss albino mice ♂	Pb nitrate	50 mg/kg b.wt.	OG, 30 d	Garlic	OG, 80 mg/kg b.wt., from the 12th d of the experiment	↓ALT_LG_ ↓AST_LG_	[235]
Swiss albino mice ♂	Pb nitrate	50 mg/kg b.wt.	OG, 30 d	Garlic	OG, 50 mg/kg b.wt., from the 12th d of the experiment	↓LPO_K_ ↗CAT_K_, ↗GPx_K_ ↑SOD_K_, ↑GST_K_, ↑GSH_K_ ↓AST_K_, ↓ALT_K_ ↑TPC_K_	[236]
Swiss albino mice ♂	Pb nitrate	50 mg/kg b.wt.	OG, 30 d	Garlic	OG, 80 mg/kg b.wt., from the 12th d of the experiment	↓LPO_K_ ↗CAT_K_, ↗GPx_K_ ↑SOD_K_, ↑GST_K_, ↑GSH_K_ ↓AST_K_, ↓ALT_K_ ↑TPC_K_	[236]
Swiss albino mice ♂	Pb nitrate	50 mg/kg b.wt.	OG, 30 d	Garlic	OG, 50 mg/kg b.wt., from the 12th d of the experiment	↙ LPO_L_ ↗SOD_L_, ↑CAT_L_, ↗GPx_L_, ↗GST_L_, ↑GSH_L_ ↓AST_L_, ↓ALT_L_, ↓ALP_L_ Normalization of TPC_L_ ↙TCC_L_	[237]
Swiss albino mice ♂	Pb nitrate	50 mg/kg b.wt.	OG, 30 d	Garlic	OG, 80 mg/kg b.wt., from the 12th d of the experiment	↙LPO_L_ ↗SOD_L_, ↑CAT_L_, ↗GPx_L_, ↗GST_L_, ↑GSH_L_ ↓AST_L_, ↓ALT_L_, ↓ALP_L_ Normalization of TPC_L_ ↙TCC_L_	[237]
Swiss albino mice ♂	Pb nitrate	50 mg/kg b.wt.	OG, 30 d	Garlic	OG, 50 mg/kg b.wt., from the 12th d of the experiment	↑Sperm concentration and motility ↓Testosterone_S_ Reduction in testicular histopathological changes	[213]
Swiss albino mice ♂	Pb nitrate	50 mg/kg b.wt.	OG, 30 d	Garlic	OG, 80 mg/kg b.wt., from the 12th d of the experiment	↑Sperm concentration and motility ↓Testosterone_S_ Reduction in testicular histopathological changes	[213]
Swiss albino mice ♂	Pb nitrate	50 mg/kg b.wt.	OG, 30 d	Garlic	OG, 50 mg/kg b.wt., from the 12th d of the experiment	↓NF-κB_LG_ ↓TNF-α_LG_, ↗IFN- γ_LG_ ↓IL-6_LG_, ↑IL-10_LG_	[238]
Swiss albino mice ♂	Pb nitrate	50 mg/kg b.wt.	OG, 30 d	Garlic	OG, 80 mg/kg b.wt., from the 12th d of the experiment	↓NF-κB_LG_ ↓TNF-α_LG_, ↑IFN- γ_LG_ ↓IL-6_LG_, ↑IL-10_LG_	[238]
Swiss albino mice ♂	Pb nitrate	50 mg/kg b.wt.	OG, 30 d	Garlic	OG, 50 mg/kg b.wt., from the 12th d of the experiment	↙LPO_T_ ↗SOD_T_, ↑CAT_T_, ↑GPx_T_ ↑GST_T_, ↗GSH_T_	[239]
Swiss albino mice ♂	Pb nitrate	50 mg/kg b.wt.	OG, 30 d	Garlic	OG, 80 mg/kg b.wt., from the 12th d of the experiment	↓LPO_T_ ↑SOD_T_, ↑CAT_T_, ↑GPx_T_, ↑GST_T_, ↑GSH_T_	[239]
Swiss albino mice ♂	Pb nitrate	50 mg/kg b.wt	OG, 30 d	Garlic	OG, 50 mg/kg b.wt., from the 12th d of the experiment	↓NF-κB_K_ ↓TNF-α_K_, ↓IFN-γ_K_, ↓IL-6_K_ ↑IL-10_K_, ↓NO_K_	[240]
Swiss albino mice ♂	Pb nitrate	50 mg/kg b.wt	OG, 30 d	Garlic	OG, 80 mg/kg b.wt., from the 12th d of the experiment	↓NF-κB_K_ ↓TNF-α_K_, ↓IFN-γ_K_, ↓IL-6_K_ ↑IL-10_K_, ↓NO_K_	[240]
Swiss albino mice ♂	Pb nitrate	50 mg/kg b.wt	OG, 30 d	Garlic	OG, 50 mg/kg b.wt., from the 12th d of the experiment	↙Chol_C_, ↙LDL_C_ ↗IFN-γ_C_, ↗IL-10_C_, ↙TNF-α_C_, ↙IL-6_C_	[241]
Swiss albino mice ♂	Pb nitrate	50 mg/kg b.wt	OG, 30 d	Garlic	OG, 80 mg/kg b.wt., from the 12th d of the experiment	↓Chol_C_, ↓LDL_C_, ↑HDL_C_ ↑IFN-γ_C_, ↑IL-10_C_ ↓TNF-α_C_, ↓IL-6_C_ Reduction in cardiac histopathological changes	[241]
Swiss albino mice ♂	Pb nitrate	50 mg/kg b.wt	OG, 30 d	Garlic	OG, 50 mg/kg b.wt., from the 12th d of the experiment	↓Pb_L_ ↓AST_S_, ↓ALT_S_, ↓ALP_S_, ↓GGT_S_ ↓NF-κB_L_, ↓TNF-α_L_, ↓IL-6_L_ ↗IL-10_L_, ↑IFN-γ_L_ ↓Caspase-3_L_, ↓Bax_L_, ↑Bcl-2_L_ Reduction in hepatic histopathological changes	[242]
Swiss albino mice ♂	Pb nitrate	50 mg/kg b.wt	OG, 30 d	Garlic	OG, 80 mg/kg b.wt., from the 12th d of the experiment	↓Pb_L_ ↓AST_S_, ↓ALT_S_, ↓ALP_S_, ↓GGT_S_ ↓NF-κB_L_, ↓TNF-α_L_, ↓IL-6_L_, ↑IL-10_L_, ↑IFN-γ_L_ ↓Caspase-3_L_, ↓Bax_L_, ↑Bcl-2_L_ Reduction in hepatic histopathological changes	[242]

b.wt.: body weight; d: days; dw: drinking water; i.p.: intraperitoneally; OG: oral gavage; p.o.: per os; wk: weeks. αKGDH: alpha-keto glutarate dehydrogenase; Chol_B_: blood cholesterol; HDL_B_: blood high density lipoprotein; Pb_B_: blood lead level; LDL_B_: blood low density lipoprotein; MDA_B_: blood malondialdehyde; TG_B_: blood triglicerydes; Pb_Bone_: bone lead level; SOD_B_: blood superoxide dismutase; BUN: blood urea nitrogen; AChE_BR_: brain acetylcholinesterase; ALT_BR_: brain alanine aminotransferase; ALP_BR_: brain alkaline phosphatase; AST_BR_: brain aspartate aminotransferase; CAT_BR_: brain catalase; Chol_BR_: brain cholesterol; Pb_BR_: brain lead level; LPP_BR_: brain lipid peroxidation products; MDA_BR_: brain malondialdehyde; ROS_BR_: brain reactive oxygen species; GSH_BR_: brain reduced glutathione; SOD_BR_: brain superoxide dismutase; TBARS_BR_: brain thiobarbituric acid reactive substances; TP_BR_: brain total protein; TPC_ContBR_: brain total protein carbonyl content; CAT_C_: cardiac catalase; Chol_C_: cardiac cholesterol; Col_ContC_: cardiac collagen content; Cu/Zn-SOD_C_: cardiac Cu/Zn-superoxide dismutase; GPx_C_: cardiac glutathione peroxidase; GR_C_: cardiac glutathione reductase; HDL_C_: cardiac high density lipoprotein; IFN-γ_C_: cardiac interferon-gamma; IL-6_C_: cardiac interleukin 6; IL-10_C_: cardiac interleukin 10; ICDH_C_: cardiac isocitrate dehydrogenase; Pb_C_: cardiac lead level; LPO_C_: cardiac lipid peroxidation; LDL_C_: cardiac low density lipoprotein; GSH_C_: cardiac reduced glutathione; SCDH_C_: cardiac succinate dehydrogenase; SOD_C_: cardiac superoxide dismutase; TBARS_C_: cardiac thiobarbituric acid reactive substances; TP_C_: cardiac total protein; TG_C_: cardiac triglicerydes; TNF-α_C_: cardiac tumor necrosis factor α; XO_C_: cardiac xanthine oxidase; CAT_RBC_: erythrocyte catalase; GSH/GSSG_RBC_: erythrocyte glutathione/glutathione disulphide ratio; H_2_O_2RBC_: erythrocyte hydrogen peroxide; MDA_RBC_: erythrocyte malondialdehyde; SOD_RBC_: erythrocyte superoxide dismutase; T-SH_ContRBC_: erythrocyte total thiol content; Pb_F_: femoral lead level; Hb: hemoglobin; ALT_K_: kidney alanine aminotransferase; ALP_K_: kidney alkaline phosphatase; AST_K_: kidney aspartate aminotransferase; CAT_K_: kidney catalase; Chol_K_: kidney cholesterol; GPx_K_: kidney glutathione peroxidase; GR_K_: kidney glutathione reductase; GST_K_: kidney glutathione S-transferase; IFN-γ_K_: kidney interferon-gamma; IL-6_K_: kidney interleukin 6; IL-10_K_: kidney interleukin 10; Pb_K_: kidney lead level; LPO_K_: kidney lipid peroxidation; LPP_K_: kidney lipid peroxidation products; MDA_K_: kidney malondialdehyde; NO _K_: kidney nitric oxide; NF-κB_K_: kidney nuclear factor-κB; ROS_K_: kidney reactive oxygen species; GSH_K_: kidney reduced glutathione; SOD_K_: kidney superoxide dismutase; TBARS_K_: kidney thiobarbituric acid reactive substances; TP_K_: kidney total protein; TPC_ContK_: kidney total protein carbonyl content; TPC_K_: kidney total protein content; TG_K_: kidney triglicerydes; TNF-α_K_: kidney tumor necrosis factor α; LtESPRs: left epidydymal sperm reserves; LtTSPR: left testicular sperm reserves; ACPT_L_: liver acid phosphatase; ALT_L_: liver alanine aminotransferase; ALP_L_: liver alkaline phosphatase; AST_L_: liver aspartate aminotransferase; Bax_L_: liver bax protein; Bcl-2_L_: liver B-cell lymphoma 2 protein; Caspase-3_L_: liver caspase-3; CAT_L_: liver catalase; Chol_L_: liver cholesterol; GPx_L_: liver glutathione peroxidase; GR_L_: liver glutathione reductase; GST_L_: liver glutathione S-transferase; HDL_L_: liver high density lipoprotein; IFN-γ_L_: liver interferon-gamma; IL-6_L_: liver interleukin 6; IL-10_L_: liver interleukin 10; LDH_L_: liver lactate dehydrogenase; Pb_L_: liver lead level; LPO_L_: liver lipid peroxidation; LPP_L_: liver lipid peroxidation products; LDL_L_: liver low density lipoprotein; MDA_L_: liver malondialdehyde; NF-κB_L_: liver nuclear factor-κB; ROS_L_: liver reactive oxygen species; GSH_L_: liver reduced glutathione; SOD_L_: liver superoxide dismutase; TBARS_L_: liver thiobarbituric acid reactive substances; TCC_L_: liver total cholesterol content; TP_L_: liver total protein; TPC_ContL_: liver total protein carbonyl content; TPC_L_: liver total protein content; TG_L_: liver triglicerydes; TNF-α_L_: liver tumor necrosis factor α; XO_L_: liver xanthine oxidase; ALT_LG_: pulmonal alanine aminotransferase; AST_LG_: pulmonal aspartate aminotransferase; Pb_Lung_: pulmonal lead level; MCV: mean corpuscular volume; Pb_M:_ muscle lead level; p53: p53 protein; PCV: packed cell volume; Pb_PL_: placenta lead level; FSH_P_: plasma follicle stimulating hormone level; LH_P_: plasma luteinizing hormone; Testosterone_P_: plasma testosterone; PFC: primary follicular count; CAT_LG_: pulmonal catalase; Chol_LG_: pulmonal cholesterol; GPx_LG_: pulmonal glutathione peroxidase; IFN-γ_LG_: pulmonal interferon-gamma; IL-6_LG_: pulmonal interleukin 6; IL-10_LG_: pulmonal interleukin 10; LDH_LG_: pulmonal lactate dehydrogenase; NF-κB_LG_: pulmonal nuclear factor-κB; GSH_LG_: pulmonal reduced glutathione; SOD_LG_: pulmonal superoxide dismutase; TPC_ContLG_: pulmonal total protein carbonyl content; TNF-α_LG_: pulmonal tumor necrosis factor α; RBC: red blood cell count; RtESPRs: right epididymal sperm reserves; RtTSPR: right testicular sperm reserves; AChE_S_: serum acetylcholinesterase; ALT_S_: serum alanine aminotransferase; ALB_S_: serum albumin; ALP_S_: serum alkaline phosphatase; AST_S_: serum aspartate aminotransferase; BChE_S_: serum butyrylcholinoesterase; CAT_S_: serum catalase; Chol_S_: serum cholesterol; Cre_S_: serum creatinine; GGT: gamma-glutamyltransferase; Glob_S_: serum globulin; GPx_S_: serum glutathione peroxidase; GR_S_: serum glutathione reductase; HDL_S_: serum high density lipoprotein; LDH_S_: serum lactate dehydrogenase; Pb_S_: serum lead level; LDL_S_: serum low density lipoprotein; GSH_S_: serum reduced glutathione; SOD_S_: serum superoxide dismutase; Testosterone_S_: serum testosterone; TBARS_S_: serum thiobarbituric acid reactive substances; TP_S_: serum total protein; TG_S_: serum triglicerydes; TNF-α_S_: tumor necrosis factor alpha; Urea_S:_ serum urea; UA_S_: serum uric acid; Pb_SP_: slpeen lead level; AChE_SC_: spinal cord acetylcholinesterase; S-D rats: Sprague-Dawley rats; Bax_T_: testicular bax protein; Bcl-2 _T_: testicular B-cell lymphoma 2 protein; Caspase-3_T_: testicular caspase-3; CAT_T_: testicular catalase; GPx_T_: testicular glutathione peroxidase; GST_T_: testicular glutathione S-transferase; Pb_T_: testicular lead level; LPO_T_: testicular lipid peroxidation; MDA_T_: testicular malondialdehyde; p53_T_: testicular p53 protein; GSH_T_: testicular reduced glutathione; SOD_T_: testicular superoxide dismutase; TBARS_T_: testicular thiobarbituric acid reactive substances; Pb_U_: urinary lead level; WBC: white blood cell count. ↑: increase; ↗: trend towards an increase; ↓: decrease; ↙: trend towards a decrease.

**Table 6:** Summary of the beneficial effects of vitamin C on Pb toxicity: an animal model.

Animals	Pb Compound	Pb Dose	Route/Time of Pb Administration	Antioxidants	Treatment Dose Route/Time of Treatment	Effects vs. Pb-Intoxicated Animals	Ref.
S-D rats ♂	Pb acetate	60 mg/kg b.wt	p.o., daily, 7 wk	Vit.C	p.o., 40 mg/kg b.wt, daily, 7 wk	↓Pb_S_ ↓AST_S_, ↓ALT_S_, ↓ALP_S_ ↓MDA_BR_, ↓NO_BR_ ↑GSH_BR_, ↑SOD_BR_, ↑CAT_BR_ Amelioration of hepatic damage	[153]
Ross broiler chicks ♂♀	Pb acetate	200 mg/kg	with diet, 42 d	Vit.C	with diet, 100 mg/kg, 42 d	↙Pb_L_ ↓TG_S_, ↓MDA_P_	[164]
Wistar rats (pups) ♀	Pb acetate	300 mg/L	dw, 41–43 d	Vit.C	dw, 2 g/L, 41–43 d	↑Hb ↓GPx_RBC_, ↓SOD_RBC_	[170]
Rats	Pb acetate	1 mg Pb/kg b.wt.	i.p., 4 wk	Vit.C	p.o., 100 mg/kg b.wt. in the 5th wk	↑CAT_K_ ↓MDA_L_, ↓MDA_BR_	[243]
Wistar rats ♂	Pb acetate	20 mg/kg b.wt.	i.g., 1x/d, 60 d	Vit.C	i.g., 20 mg/kg b.wt., every other d, 30 min before Pb acetate, 60 d	Reduction in hepatic/renal/cerebral/testicular histopathological changes	[206]
Albino rats ♂	Pb acetate	500 mg/kg diet	with diet, daily 2, 4, 6 wk	Vit.C Silymarin	i.g., 1 mg/100 g b.wt., 3x/wk, 2, 4, 6 wk i.g., 1 mg/100 g b.wt., 3x/wk, 2, 4, 6 wk	↓Pb_B_ Reduction in hepatic histopathological changes	[202]
Swiss albino mice ♂	Pb acetate	20 mg/kg	OG, 5 d	Vit.C	OG, 500 mg/kg, 1 h prior to Pb acetate administration, 5 d	Reduction in hepatic/splenic histopathological changes	[200]
S-D rats ♂	Pb acetate	2%	dw, 1x/d, 3 m	Vit.C	dw, 100 mg/kg b.wt., 1x/d, 3 m	↓AST_P_, ↓ALT_P_, ↓ALP_P_ ↓LOOH_B_, ↑GSH_RBC_ ↑TP_P_ ↓TG_P_, ↓Chol_P_, ↓LDL_P_, ↑HDL_P_	[149]
Wistar rats ♀	Pb acetate	1500 ppm	dw, 0–21 d of pregnancy	Vit.C	i.p., 500 mg/kg, 0–21 d of pregnancy	↓Pb_B_ in neonates ↓Pb_Bone_ in neonates Normalization of bone ossification in neonates	[172]
Albino rats ♀	Pb acetate	20 mg/kg b.wt.	p.o., daily, 10 wk	Vit.C	p.o., daily, 500 mg/kg b.wt., 10 wk	Normalization of RBC, Hb, Normalization of PCV, MCV ↓AST_S_, ↓ALT_S_ ↓Crea_S_ Reduction in hepatic/renal/cerebral/splenic histopathological changes	[139]
S-D rats ♀	Pb acetate	0.3%	dw, from gestation until 21st postnatal d	Vit.C	OG, 100 mg/kg, from gestation until 21st postnatal d	↓Pb_B_ ↓NMDAR1_BR_ Normalization of MnSOD_BR_ Prevention of reduction in Purkinje cells Attenuation of reduction in the expression of synaptophysin and axonal myelin basic protein	[212]
Wistar Rats ♂	Pb acetate	10 mg/kg b.wt.	i.p., 1x/d, 7 d	Vit.C	OG, 200 mg/kg b.wt., 1x/d, 7 d	↓MDA_BR_ ↑SOD_BR_, ↑CAT_BR_, ↑GPx_BR_ Normalization of BchE, AChE, Na^+^/K^+^-ATPase XO, EPI, DA, MAO	[194]
Rats ♀♂	Pb acetate	1 mg Pb/kg b.wt.	i.p., 30 d	Vit.C	p.o., 100 mg/kg b.wt., 7 d	↙MDA_C_	[188]
Wistar Rats ♀♂	Pb acetate	100 ppm	dw, 30 d	Vit.C	with diet, 800 mg/kg, 30 d	↓MDA_L_, MDA_K_, MDA_LG_ ↓CD_L_, ↓CD_K_, ↓CD_LG_ ↓HYPDX_L_, ↓HYPDX_K_, ↓HYPDX_LG_	[179]
Mice ♂	Pb acetate	0.2%	dw, 42 d	Vit.C Vit. B_1_ Vit.C Vit. B_1_ Vit.C Vit. B_1_	OG, 140 mg/kg b.wt., 42 d OG, 10 mg/kg b.wt., 42 d OG, 420 mg/kg b.wt., 42 d OG, 10 mg/kg b.wt., 42 d OG, 420 mg/kg b.wt., 42 d OG, 30 mg/kg b.wt., 42 d	Normalization of Hb ↑SOD_B_, GPx_B_, GSH_L_ Normalization of Hb ↑SOD_B_, GPx_B_, GSH_L_ Normalization of Hb ↑SOD_B_, GPx_B_, GSH_L_ Reduction in DNA damage in hepatocytes	[143]
Wistar-Kyoto rats ♂	Pb acetate	100 ppm	p.o., 3 m	Vit.C	p.o., 20 mg/rat/day, 3 m	↓MDA_P_ ↑TAC_B_ Normalization of urinary 8-OH-dG	[159]
S-D rats	Pb acetate	1000 ppm	dw, 4 wk	Vit.C	500 ppm, 4 wk	Normalization of Hb ↓AST_S_, ↓ALT_S_, ↓Crea_S_, ↓UA_S_ ↓MDA_S_, ↓SOD_RBC_	[244]
Albino rats	Pb acetate	35 mg/kg	i.p., daily, 3 d	Vit.C	i.p., 10 mg/kg, daily, 3 d	Normalization of Hb ↓AST_S_, ↙ALT_S_, ↙ALP_S_ ↓MDA_L_ Normalization of GSH_L_	[245]
Wistar rats ♂	Pb acetate	3 g/L	p.o., daily, 28 d	Vit.C	p.o., 500 mg/kg b.wt., daily 28 d	↓Pb_B_, ↓Pb_L_ ↓MDA_L_ ↓ALP_L_ ↑SOD_L_, ↑CAT_L_, ↑GSH_L_ Reduction in hepatic histopathological changes	[177]
Wistar rats ♀	Pb acetate	100 µmol/kg b.wt.	i.p., 7 d	Vit.C	i.p., 500 mg/kg b.wt., 7 d after Pb acetate administration	↓ALT_S_, ↓ALP_S_	[224]
Rats ♀	Pb acetate	100 µmol/kg b.wt.	i.p., 7 d	Vit.C	i.p., 500 mg/kg b.wt., 7 d after Pb acetate administration	↓Pb_BR_, ↓Pb_L_, ↓Pb_K_	[199]
S-D rats ♂	Pb acetate	60 mg/kg b.wt.	p.o., 3x/wk every other day, 6 wk	Vit.C	p.o., 40 mg/kg b.wt., 20 min after Pb acetate administration 3x/wk every other day, 6 wk	↓Pb_T_ ↓MDA_T_, ↓NO_T_ ↑GSH_T_, ↑SOD_T_, ↑CAT_T_ ↑FSH_S_, ↑Testosterone_S_ ↑Sperm motility, ↑Sperm count Improvement of sperm morphology Reduction in testicular histopathological changes	[189]
S-D rats ♀	Pb acetate	0.2%	dw, during pregnancy and lactation	Vit.C	p.o., 100 mg/kg/d, 1x/d, during pregnancy and lactation	↓Pb_B_ (dams) ↓Bax_H_ expression (pups) ↗Bcl-2_H_ expression (pups) Reduction in hippocampal histopathological changes (pups)	[198]
S-D rats ♀	Pb acetate	0.2%	dw, during pregnancy and lactation	Vit.C	p.o., 100 mg/kg/d, during pregnancy and lactation	↓Pb_B_ (dams) ↗Cu/Zn-SOD_BR_ expression level (pups) ↗Mn-SOD_BR_ expression level (pups) ↑CAT_BR_ expression level (pups) Reduction in hippocampal histopathological changes (pups)	[209]
Wistar rats ♂	Pb acetate	1 mg/kg	i.v., 35 h after vit.C administration	Vit.C	i.v., 25 mg/kg/6 h	↓Pb_F_, ↓Pb_K_, ↓Pb_L_ ↓t_1/2_Pb_F_, ↓t_1/2_Pb_P_	[246]
S-D rats ♂	Pb acetate	10 mg/kg b.wt.	i.p., 6 wk	Vit.C	dw, 500 mg/L, 6 wk	↓ROS_S_ Prevention of SOPR	[218]
Swiss albino mice ♂	Pb acetate	10 mg/kg b.wt.	i.p., 8 wk	Vit.C	i.p., 10 mg/kg b.wt., 8 wk	↓MDA_T_ ↑Sperm count ↓Sperm abnormality	[247]
Wistar rats ♂	Pb acetate	0.1%	dw, 3 m	Vit.C	p.o., 25 mg/kg, 5 d	↓Pb_B_, ↙Pb_L_, ↙Pb_K_, ↙Pb_BR_ ↓TBARS_L_, ↓GSSG_L_, ↑CAT_L_ ↙TBARS_K_, ↓CAT_BR_	[162]
Mice ♂	Pb acetate	0.2%	dw, 42 d	Vit.C	OG, 140 mg/kg b.wt., 42 d	↙ Expression of caspase-3_T_ ↙ Expression of TGFβ_T_ ↓DNA damage in testes	[248]
Mice ♂	Pb acetate	0.2%	dw, 42 d	Vit.C	OG, 420 mg/kg b.wt., 42 d	↓Expression of caspase-3_T_ ↓Expression of TGF-β_T_ ↓DNA damage in testes	[248]
Albino rats ♀	Pb acetate	0.1 mg/L	dw, 10 wk	Vit.C	dw, 1 mg/L, 10 wk	↓MDA_S_ Reduction in hepatic histopathological changes	[201]
Albino rats ♂	Pb acetate	20 mg/kg b.wt.	OG, 8 wk	Vit.C	OG, 20 mg/kg b.wt., 8 wk	↓MDA_T_ ↑GSH_T_, ↑CAT_T_ ↑Sperm count, ↑Sperm viability ↑Testosterone_S_	[249]
S-D rats ♂	Pb acetate	0.1%	dw, 10 wk	Vit.C	p.o., 350 mg/rat/day, 10 wk	↙Pb_Bone_ ↙Pb_K_, ↙Pb_L_, ↙Pb_BR_	[250]
Wistar rats ♂	Pb acetate	500 ppm	in diet, 16 d	Vit.C	in diet, 1%, 16 d	↙Pb_L_	[251]
Wistar rats ♂	Pb acetate	500 ppm	in diet, 19 d	Vit.C	in diet, 1%, 19 d	↓Pb_K_, ↙Pb_L_, ↓Pb_Bone_ Normalization of Hb	[251]
Wistar rats ♂	Pb acetate	500 ppm	in diet, 56 d	Vit.C	in diet, 1%, 56 d	↙Pb_L_, ↓Pb_K_, ↓Pb_Bone_ Normalization of Hb	[252]
Albino rats ♂	Pb acetate	10 mg Pb/4 mL/kg b.wt.	OG, 6 d/wk 8 wk	Vit.C	OG, 25 mg/4 mL/kg b.wt., 6 d/wk, 8 wk	↓Pb_L_, ↓Pb_K_, ↓Pb_B_	[253]

b.wt.: body weight; d: days; dw: drinking water; i.p.: intraperitoneally; i.v.: intravenously; OG: oral gavage; p.o.: per os; wk: weeks. AChE: acetylcholinesterase; GPx_B_: blood glutathione peroxidase; Pb_B_: blood lead level; LOOH_B_: blood lipid hydroxyperoxide; SOD_B_: blood superoxide dismutase; TAC_B_: blood total antioxidant capacity; Pb_Bone_: bone lead level; CAT_BR_: brain catalase; Cu/Zn-SOD_BR_: brain Cu/Zn-superoxide dismutase; NMDAR1_BR_: brain glutamatergic N-methyl-d-aspartate receptor subtype 1; GPx_BR_: brain glutathione peroxidase; Pb_BR_: brain lead level; MDA_BR_: brain malondialdehyde; Mn-SOD_BR_: brain manganese superoxide dismutase; NO_BR_: brain nitric oxide; GSH_BR_: brain reduced glutathione; SOD_BR_: brain superoxide dismutase; BChE: butyrylcholinoesterase; MDA_C_: cardiac malondialdehyde; DA: dopamine; EPI: epinephrine; GPx_RBC_: erythrocyte glutathione peroxidase; GSH_RBC_: erythrocyte reduced glutathione; SOD_RBC_: erythrocyte superoxide dismutase; Pb_F_: femoral lead level; t_1/2_Pb_F_: half-life of lead in femur; t_1/2_Pb_P_: half-life of lead in plasma; Hb: hemoglobin; 8-OH-dG: 8-hydroxy-2-deoxyguanosine; Bcl-2_H_: hippocampal B-cell lymphoma 2 protein; Bax_H_: hippocampal bax protein; CAT_K_: kidney catalase; CD_K_: kidney conjugated dienes; HYPDX_K_: kidney hydroperoxide level; Pb_K_: kidney lead level; MDA_K_: kidney malondialdehyde; TBARS_K_: kidney thiobarbituric acid reactive substances; ALP_L_: liver alkaline phosphatase; CAT_L_: liver catalase; CD_L_: liver conjugated dienes; HYPDX_L_: liver hydroperoxide level; Pb_L_: liver lead level; MDA_L_: liver malondialdehyde; GSSG_L_: liver oxidixed glutathione; GSH_L_: liver reduced glutathione; SOD_L_: liver superoxide dismutase; TBARS_L_: liver thiobarbituric acid reactive substances; MCV: mean corpuscular volume; MAO: monoamine oxidase; PCV: packed cell volume; ALT_P_: plasma alanine aminotransferase; ALP_P_: plasma alkaline phosphatase; AST_P_: plasma aspartate aminotransferase; Chol_P_: plasma cholesterol; HDL_P_: plasma high density lipoprotein; LDL_P_: plasma low density lipoprotein; MDA_P_: plasma malondialdehyde; TP_P_: plasma total protein; TG_P_: plasma triglicerydes; CD_LG_: pulmonal conjugated dienes; HYPDX_LG_: pulmonal hydroperoxide level; MDA_LG_: pulmonal malondialdehyde; RBC: red blood cell count; ALT_S_: serum alanine aminotransferase; ALP_S_: serum alkaline phosphatase; AST_S_: serum aspartate aminotransferase; Crea_S_: serum creatinine; FSH_S_: serum follicle stimulating hormone level; Pb_S_: serum lead level; MDA_S_: serum malondialdehyde; ROS_S_: serum reactive oxygen species; TG_S_: serum triglicerydes; Testosterone_S_: serum testosterone; UA_S_: serum uric acid; Na^+^/K^+^- sodium/potassium adenosine triphosphatase; SOPR: sperm-oocyte penetration rate; S-D rats: Sprague-Dawley rats; Caspase-3_T_: testicular caspase-3; CAT_T_: testicular catalase; Pb_T_: testicular lead level; MDA_T_: testicular malondialdehyde; NO_T_: testicular nitric oxide; GSH_T_: testicular reduced glutathione; TGF-β_T_: transforming growth factor-beta; SOD_T_: testicular superoxide dismutase; XO: xanthine oxidase. ↑: increase; ↗: trend towards an increase; ↓: decrease; ↙: trend towards a decrease.

**Table 7:** Summary of the beneficial effects of vitamin E on Pb toxicity: an animal model.

Animals	Pb Compound	Pb Dose	Route/Time of Pb Administration	Antioxidants	Treatment Dose Route/Time of Treatment	Effects vs. Pb-Intoxicated Animals	Ref.
Wistar rats ♂	Pb acetate	1000 ppm	OG, 35 d	Vit.E	in chow, 300 mg/kg of chow, 35 d	↓MDA_T_ ↑SOD_T_ ↑Sperm motility and viability	[222]
S-D rats ♂	Pb acetate	60 mg/kg b.wt	p.o., daily, 7 wk	Vit.E	p.o., 150 mg/kg b.wt., 7 wk	↓Pb_S_ ↓AST_S_, ↓ALT_S_, ↓ALP_S_ ↓MDA_BR_, ↓NO_BR_ ↑GSH_BR_, ↑SOD_BR_, ↑CAT_BR_ Amelioration of hepatic damage	[153]
Rats	Pb acetate	1 mg/kg b.wt.	i.p., 4 wk	Vit.E	p.o., 100 mg/kg b.wt., in the 5th wk	↓MDA_L_, ↓MDA_BR_	[243]
S-D albino rats ♂	Pb acetate	10 mg/kg/d	dw, 1 m	Vit.E	p.o., 100 mg/kg b.wt., 1 m	↓Pb_S_ Normalization of Hb, RBC, WBC ↓Chol_S_, ↓Chol_L_, ↓Chol_C_, ↓Chol_K_ ↓LDL_S_, ↑HDL_S_ ↓TG_S_, ↓TG_L_, ↓TG_C_, ↓TG_K_ ↓TBARS_C_, ↓TBARS_K_, ↓TBARS_L_, ↓TBARS_S_ ↑GSH_C_, ↑GSH_K_, ↑GSH_L_, ↑GSH_S_ ↑CAT_C_, ↑CAT_K_, ↑CAT_L_, ↑CAT_S_ ↑SOD_C_, ↑SOD_K_, ↑SOD_L_, ↑SOD_S_ ↑GR_C_, ↑GR_K_, ↑GR_L_, ↑GR_S_ ↑GPx_C_, ↑GPx_K_, ↑GPx_L_, ↑GPx_S_ Reduction in hepatic/renal histopathological changes	[168]
S-D rats ♂	Pb acetate	2%	dw, daily, 3 m	Vit.E	p.o., 100 mg/kg b.wt., daily, 3 m	↓AST_P_, ↓ALT_P_, ↓ALP_P_ ↓LOOH_B_, ↑GSH_RBC_ ↑TP_P_ ↓TG_P_, ↓Chol_P_, ↓LDL_P_, ↑HDL_P_	[149]
Rats ♂♀	Pb acetate	1 mg/kg b.wt.	i.p., 30 d	Vit.E	p.o., 100 mg/kg b.wt., 7 d	↙Pb_B_, ↙Pb_C_ ↙MDA_C_	[188]
Wistar Rats ♀♂	Pb acetate	100 ppm	dw, 30 d	Vit.E	with diet, 50 IU/kg, 30 d	↓MDA_L_, ↓MDA_K_, ↓MDA_LG_ ↓ConD_L_, ↓ConD_LG_, ↓ConD_K_ ↓HYPDX_L_, ↓HYPDX_LG_, ↓HYPDX_K_	[179]
S-D rats ♂	Pb acetate	60 mg/kg b.wt.	p.o., 3x/wk every other day, 6 wk	Vit.E	p.o., 150 mg/kg b.wt., 20 min after Pb acetate administration 3x/wk, 6 wk	↓Pb_T_ ↓MDA_T_, ↓NO_T_ ↑GSH_T_, ↑SOD_T_, ↑CAT_T_ ↑FSH_S_, ↑testosterone_S_ ↑Sperm motility, ↑Sperm count Improvement of sperm morphology Reduction in testicular histopathological changes	[189]
S-D rats	Pb acetate	1000 ppm	dw, 4 wk	Vit.E	20 ppm, 4 wk	Normalization of Hb ↓MDA_S_ ↓ALT_S_, ↓AST_S_, ↓Urea_S_, ↓Crea_S_	[244]
S-D rats ♀	Pb acetate	160 mg/L	dw, 1 m	Vit.E	p.o., 100 mg/kg/d, 1 m	↓Pb_B_ Normalization of Hb ↓ALT_S_, ↓AST_S_ ↓TBARS_C_, ↓TBARS_K_, ↓TBARS_L_, ↓TBARS_S_ ↑TP_S_, ↑TP_L_, ↑TP_C_ ↓Chol_S_, ↓TG_S_, ↓LDL_S_ ↓Chol_C_, ↓TG_C_, ↓LDL_C_ ↓Chol_L_, ↓TG_L_, ↓LDL_L_, ↑HDL_L_	[144]
Wistar rats ♂♀	Pb acetate	10 mg/kg/d	i.p., 5x/wk, 6 wk	Vit.E	OG, 50 mg/kg/d, 5x/wk, 6 wk	↓MDA_P_ ↑TAC_P_ ↓Caspase-3_P_ ↑Gonadotrophic/gonadal hormones in the plasma Reduction in gonadal histopathological changes	[166]
Rats ♂	Pb acetate	10 mg/kg b.wt.	i.p., 5 d/wk, 3 m	Vit.E Vit.E Vit.E	i.p., 100 mg/kg b.wt., 12 h before Pb acetate injection, 1 m i.p., 100 mg/kg b.wt., 12 h before Pb acetate injection, 2 m i.p., 100 mg/kg b.wt., 12 h before Pb acetate injection, 3 m	Reduction in testicular histopathological changes Reduction in testicular histopathological changes Reduction in testicular histopathological changes	[216]
S-D rats ♂	Pb chloride	12 mg/kg	i.p., 24 d (from 21st to 45th d of experiment)	Vit.E	i.p., 50 IU/kg b.wt., 45 d	↓LPO_T_ ↑SOD_T_, ↑GPx_T_, ↑CAT_T_ ↑LH_S_, ↑FSH_S_ ↑Testosterone_S_ ↑Sperm motility and viability ↑Sperm count	[192]
Albino rats ♂	Pb acetate	1.5 g/L	dw, 8 wk	Vit.E	p.o., 600 mg/kg b.wt., 3x/wk, 8 wk	↓Pb_T_ ↑GSH_T_, ↑GST_T_, ↓MDA_T_ ↑Testosterone_S_ ↑Sperm viability ↓Sperm abnormality Reduction in testicular histopathological changes	[219]
Wistar rats ♂	Pb acetate	20 mg/kg b.wt.	i.p., 20 d	Vit.E	p.o., 600 mg/kg/rat, 20 d	↑FSH_S_, LH_S_ ↑Testosterone_S_ ↑Sperm count, ↑Sperm motility ↓Sperm abnormality Reduction in chromosomal aberrations in bone marrow cells Reduction in testicular histopathological changes	[214]
Wistar rats ♂	Pb acetate	500 ppm	dw, 15 d	Vit.E	i.g., 1.7 mg/kg b.wt., 15 d	↓TBARS_B_ ↑ALAD_B_	[254]
Wistar rats ♂	Pb acetate	500 ppm	dw, 30 d	Vit.E	i.g., 1.7 mg/kg b.wt., 30 d	↓TBARS_B_ ↑ALAD_B_	[254]
Wistar rats ♂	Pb acetate	0.2%	dw, 1x/d, 3 m	Vit.E	OG, 150 mg/kg, 1x/d, 3 m	↓MDA_S_ ↑TAC_S_, ↓TOS_S_	[167]
Wistar rats ♂	Pb acetate	0.2%	dw, 1x/d, 2 m	Vit.E	OG, 150 mg/kg, 1x/d, 1 m after Pb administration	↓MDA_S_ ↑TAC_S_, ↓TOS_S_	[167]
Wistar rats ♂	Pb acetate	0.2%	dw, 1x/d, 2 m	Vit.E	OG, 150 mg/kg, 1x/d, 1 m before Pb administration	↓MDA_S_ ↑TAC_S_, ↓TOS_S_	[167]
Wistar rats ♂	Pb acetate	0.2%	OG, 30 d	Vit.E	OG, 50 μg/rat, 30 d	↑STL, ↓TDC ↓Impairment of learning	[255]
S-D rats ♂	Pb acetate	10 mg/kg b.wt.	i.p., 6 wk	Vit.E	with diet, 150 mg/kg chow, 6 wk	↓ROS_S_ Prevention of SOPR	[218]
Swiss mice ♂	Pb acetate	0.5 mg/kg b.wt.	i.p.,1x/d, 30 d	Vit.E	i.m., 5 mg/kg b.wt., alternate days, 30 d	↑T_3S_ ↑5′D-I_L_ ↓MDA_L_, ↑SOD_L_, ↑CAT_L_	[256]
TO mice ♂	Pb acetate	1 mg/kg b.wt.	i.p., 3 wk	Vit.E	i.p., 100 mg/kg b.wt., 3 wk	↑Seminiferous tubules sperm count ↑Density of spermatozoa in seminiferous tubules ↓Nuclear disorganization of spermatids in seminiferous tubules ↑Epidydymal spermatozoa ↑Epidydymal sperm count	[257]
Wistar rats ♂	Pb acetate	0.1%	dw, 3 m	Vit.E	i.m., 5 mg/kg b.wt., 1x/d, 5 d	↓Pb_B_, ↙Pb_L_, ↙Pb_K_, ↙Pb_BR_ ↓TBARS_L_, ↓GSSG_L_, ↑CAT_L_ ↓TBARS_K_, ↓GPx_K_ ↓CAT_BR_	[258]
Albino rats ♀	Pb acetate	0.1 mg/L	dw, 10 wk	Vit.E	1000 IU/kg diet, 10 wk	↓MDA_S_ Reduction in hepatic histopathological changes	[201]
Swiss albino mice ♂	Pb acetate	10 mg/kg b.wt.	i.p., 8 wk	Vit.E	i.p., 100 mg/kg b.wt., 8 wk	↓MDA_T_ ↑Sperm count ↓Sperm abnormality	[247]

b.wt.: body weight; d: days; dw: drinking water; i.p.: intraperitoneally; i.v.: intravenously; OG: oral gavage; p.o.: per os; wk: weeks. ALAD_B_: blood delta-aminolevulinic acid dehydratase; Pb_B_: blood lead level; LOOH_B_: blood lipid hydroxyperoxide; TBARS_B_: blood thiobarbituric acid reactive substances; CAT_BR_: brain catalase; Pb_BR_: brain lead level; MDA_BR_: brain malondialdehyde; NO_BR_: brain nitric oxide; GSH_BR_: brain reduced glutathione; SOD_BR_: brain superoxide dismutase; CAT_C_: cardiac catalase; Chol_C_: cardiac cholesterol; GPx_C_: cardiac glutathione peroxidase; GR_C_: cardiac glutathione reductase; Pb_C_: cardiac lead level; LDL_C_: cardiac low density lipoprotein; MDA_C_: cardiac malondialdehyde; GSH_C_: cardiac reduced glutathione; SOD_C_: cardiac superoxide dismutase; TP_C_: cardiac total protein; TBARS_C_: cardiac thiobarbituric acid reactive substances; TG_C_: cardiac triglicerydes; GSH_RBC_: erythrocyte reduced glutathione; Hb: hemoglobin; CAT_K_: kidney catalase; Chol_K_: kidney cholesterol; ConD_K_: kidney conjugated dienes; GPx_K_: kidney glutathione peroxidase; GR_K_: kidney glutathione reductase; HYPDX_K_: kidney hydroperoxide level; Pb_K_: kidney lead level; MDA_K_: kidney malondialdehyde; GSH_K_: kidney reduced glutathione; SOD_K_: kidney superoxide dismutase; TBARS_K_: kidney thiobarbituric acid reactive substances; TG_K_: kidney triglicerydes; CAT_L_: liver catalase; Chol_L_: liver cholesterol; ConD_L_: liver conjugated dienes; GPx_L_: liver glutathione peroxidase; GR_L_: liver glutathione reductase; HDL_L_: liver high density lipoprotein; HYPDX_L_: liver hydroperoxide level; Pb_L_: liver lead level; LDL_L_: liver low density lipoprotein; MDA_L_: liver malondialdehyde; GSSG_L_: liver oxidized glutathione; GSH_L_: liver reduced glutathione; SOD_L_: liver superoxide dismutase; TBARS_L_: liver thiobarbituric acid reactive substances; TP_L_: liver total protein; TG_L_: liver triglicerydes; 5′D-I_L_: liver type-I iodothyronine 5′-monodeiodinase; ALT_P_: plasma alanine aminotransferase; ALP_P_: plasma alkaline phosphatase; AST_P_: plasma aspartate aminotransferase; Caspase-3_P_: plasma caspase-3; Chol_P_: plasma cholesterol; HDL_P_: plasma high density lipoprotein; LDL_P_: plasma low density lipoprotein; MDA_P_: plasma malondialdehyde; TAC_P_: plasma total antioxidant capacity; TP_P_: plasma total protein; TG_P_: plasma triglicerydes; ConD_LG_: pulmonal conjugated dienes; HYPDX_LG_: pulmonal hydroperoxide level; MDA_LG_: pulmonal malondialdehyde; RBC: red blood cell count; S-D rats: Sprague-Dawley rats; ALT_S_: serum alanine aminotransferase; ALP_S_: serum alkaline aminotransferase; AST_S_: serum aspartate aminotransferase; CAT_S_: serum catalase; Crea_S_: serum creatinine; Chol_S_: serum cholesterol; FSH_S_: serum follicle stimulatin hormone level; GPx_S_: serum glutathione peroxidase; GR_S_: serum glutathione reductase; HDL_S_: serum high density lipoprotein; Pb_S_: serum lead level; LDL_S_: serum low density lipoprotein; LH_S_: serum luteinizing hormone; MDA_S_: serum malondialdehyde; ROS_S_: serum reactive oxygen species; GSH_S_: serum reduced glutathione; SOD_S_: serum superoxide dismutase; Testosterone_S_: serum testosterone; TBARS_S_: serum thiobarbituric acid reactive substances; TAC_S_: serum total antioxidant capacity; TOS_S_: serum total oxidant status; TP_S_: serum total protein; TG_S_: serum triglicerydes; T_3S_: serum 3,3′,5-triiodothyronine; Urea_S_: serum urea; SOPR: sperm-oocyte penetration rate; STL: step through latency; CAT_T_: testicular catalase; GPx_T_: testicular glutathione peroxidase; GST_T_: testicular glutathione-S-transferase; Pb_T_: testicular lead level; LPO_T_: testicular lipid peroxidation; MDA_T_: testicular malondialdehyde; NO_T_: testicular nitric oxide; GSH_T_: testicular reduced glutathione; SOD_T_: testicular superoxide dismutase; TDC: time in the dark compartment; WBC: white blood cell count. ↑: increase; ↓: decrease; ↙: trend towards a decrease.

## Data Availability

No new data were created or analyzed in this study. Data sharing is not applicable to this article.
